# Association Between Sociodemographic Factors and Vaccine Acceptance for Influenza and SARS-CoV-2 in South Korea: Nationwide Cross-Sectional Study

**DOI:** 10.2196/56989

**Published:** 2024-11-01

**Authors:** Seohyun Hong, Yejun Son, Myeongcheol Lee, Jun Hyuk Lee, Jaeyu Park, Hayeon Lee, Elena Dragioti, Guillaume Fond, Laurent Boyer, Guillermo Felipe López Sánchez, Lee Smith, Mark A Tully, Masoud Rahmati, Yong Sung Choi, Young Joo Lee, Seung Geun Yeo, Selin Woo, Dong Keon Yon

**Affiliations:** 1 Department of Medicine, Kyung Hee University College of Medicine, Seoul, Republic of Korea; 2 Center for Digital Health, Medical Science Research Institute, Kyung Hee University College of Medicine, 23 Kyungheedae-ro, Dongdaemun-gu, Seoul, 02447, Republic of Korea, 82 2 6935 2476, 82 504 478 0201; 3 Department of Precision Medicine, Kyung Hee University College of Medicine, Seoul, Republic of Korea; 4 Department of Regulatory Science, Kyung Hee University, Seoul, Republic of Korea; 5 Health and Human Science, University of Southern California, Los Angeles, CA, United States; 6 Research Laboratory Psychology of Patients, Families, and Health Professionals, Department of Nursing, School of Health Sciences, University of Ioannina, Ioannina, Greece; 7 CEReSS-Health Service Research and Quality of Life Center, Assistance Publique-Hôpitaux de Marseille, Aix-Marseille University, Marseille, France; 8 Division of Preventive Medicine and Public Health, Department of Public Health Sciences, University of Murcia School of Medicine, Murcia, Spain; 9 Centre for Health, Performance and Wellbeing, Anglia Ruskin University, Cambridge, United Kingdom; 10 School of Medicine, Ulster University, Londonderry, Northern Ireland, United Kingdom; 11 Department of Physical Education and Sport Sciences, Faculty of Literature and Human Sciences, Lorestan University, Khoramabad, Iran; 12 Department of Physical Education and Sport Sciences, Faculty of Literature and Humanities, Vali-E-Asr University of Rafsanjan, Rafsanjan, Iran; 13 Department of Pediatrics, Kyung Hee University Medical Center, Kyung Hee University College of Medicine, Seoul, Republic of Korea; 14 Department of Obstetrics and Gynecology, Kyung Hee University College of Medicine, Seoul, Republic of Korea; 15 Department of Otolaryngology-Head and Neck Surgery, Kyung Hee University Medical Center, Kyung Hee University College of Medicine, Seoul, Republic of Korea

**Keywords:** COVID-19, influenza, South Korea, vaccination, vaccinations, COVID-19 vaccine, COVID-19 vaccination, SARS-CoV-2, SARS-CoV-2 vaccine, pandemic, SARS-CoV-2 vaccination, vaccine, vaccine hesitancy

## Abstract

**Background:**

The imperative arises to study the impact of socioeconomic factors on the acceptance of SARS-CoV-2 and influenza vaccines amid changes in immunization policies during the COVID-19 pandemic.

**Objective:**

To enhance targeted public health strategies and improve age-specific policies based on identified risk factors, this study investigated the associations between sociodemographic factors and vaccination behaviors during the COVID-19 pandemic, with emphasis on age-specific vaccine cost policies.

**Methods:**

This study analyzed data from the Korean Community Health Survey 2019‐2022 with 507,964 participants to investigate the impact of age-specific policies on vaccination behaviors during the pandemic period. Cohorts aged 19‐64 years and 65 years or older were stratified based on age (years), sociodemographic factors, and health indicators. The cohorts were investigated to assess the influence of relevant risk factors on vaccine acceptance under the pandemic by using weighted odds ratio and ratio of odds ratio (ROR).

**Results:**

Among 507,964 participants, the acceptance of the SARS-CoV-2 vaccine (COVID-19 vaccine) was higher among individuals with factors possibly indicating higher socioeconomic status, such as higher education level (age 19‐64 years: ROR 1.34; 95% CI 1.27‐1.40 and age ≥65 years: ROR 1.19; 95% CI 1.01‐1.41) and higher income (age 19‐64 years: ROR 1.67; 95% CI 1.58‐1.76 and age ≥65 years: ROR 1.21; 95% CI 1.06‐1.38) for both age cohorts compared to influenza vaccine acceptance before the pandemic. In the context of influenza vaccination during the pandemic, the older cohort exhibited vaccine hesitancy associated with health care mobility factors such as lower general health status (ROR 0.89; 95% CI 0.81‐0.97).

**Conclusions:**

SARS-CoV-2 vaccination strategies should focus on reducing hesitancy among individuals with lower social participation. To improve influenza vaccine acceptance during the pandemic, strategies for the younger cohort should focus on individuals with lower social participation, while efforts for the older cohort should prioritize individuals with limited access to health care services.

## Introduction

Understanding the interaction between age and vaccination policies is imperative for effective global public health [1]. In South Korea, this is notably observed in the provision of influenza vaccines, which are free of charge for individuals aged 65 years or older, owing to their heightened susceptibility due to advanced age [2-4]. In contrast, individuals within the age cohort of 19 to 64 years bear the financial responsibility for procuring the influenza vaccine [5]. However, the landscape of vaccine accessibility underwent a profound transformation during the COVID-19 pandemic, caused by the SARS-CoV-2 virus. Influenced by the pandemic, universal access to SARS-CoV-2 vaccines (COVID-19 vaccines) was extended to all demographic strata [6,7]. While many studies have analyzed which socioeconomic factors are associated with vaccination for influenza and SARS-CoV-2, they often do not provide information on how age-specific policy differences and pandemic conditions affect vaccination uptake [8]. Given the differences in how different age groups access vaccines, the need to examine socioeconomic factors influencing vaccination acceptance during pandemics, dependent upon age and distinct vaccination strategies, is paramount [9].

In this comprehensive study, we conducted a large-scale population-based study to investigate socioeconomic factors associated with the acceptance of SARS-CoV-2 and influenza vaccines among the adult population in South Korea [10]. Our focus extends across both before and during the COVID-19 pandemic periods. In South Korea, individuals aged 65 years and older are classified as older populations and are considered particularly vulnerable, prompting targeted governmental support in the form of free influenza and SARS-CoV-2 vaccinations to address their specific health risks and needs. To delineate disparities in vaccination acceptance based on age-specific policies, we categorized the study population into 2 distinct age groups: younger (19-64 years) and older (≥65 years) populations. In examining the younger cohort, we investigated the difference between paid and free vaccines and how the pandemic affected them; for the older populations, who had access to free vaccines both before and after the pandemic, our primary focus rested on how the pandemic itself influenced vaccination behaviors.

Our objective was to understand the impact of age-specific policies and the pandemic on vaccination acceptance [11]. Among a variety of vaccines available in South Korea, influenza and SARS-CoV-2 vaccines were crucial to public health strategies during the COVID-19 pandemic. Both had similar transmission mechanisms and targeted similar demographics. Furthermore, the long-established influenza vaccination program in Korea provides robust data that enhance the reliability of comparative analyses. Our study compares an influenza vaccine that is offered for a fee to young adults and free to older adults with a SARS-CoV-2 vaccine that is offered for free to all ages [12]. By examining the impact of this vaccine policy on various socioeconomic and age-related factors, we analyzed differences in vaccine access and acceptance. We aimed to provide insights that can contribute to future public health policy development. Through this study, we can better identify demographic groups with particularly low vaccine acceptance. These groups will be characterized by specific demographic factors, and recognizing these factors will allow us to facilitate targeted public health interventions, ultimately reducing the risk of infection during future pandemics.

## Methods

### Ethical Considerations

The study protocol was approved by the Institutional Review Board of the Korean Disease Control and Prevention Agency (2010-02CON-22-P, 2011-05CON04-C, 2012-07CON-01-2C, 2013-06EXP-01-3C, 2014-08EXP-09-4CA, and 2016-10-01-TA) and by the local law of State-Approved Statistics (#117075) and Enforcement Regulation (article 2, paragraph 2, item 1) of the Bioethics and Safety Act from the Korean government. The second analysis in this study was approved by the Institutional Review Board of Kyung Hee University. Ethical considerations were upheld, adhering to the principles outlined in the Declaration of Helsinki. Furthermore, the Korean Community Health Survey (KCHS) provides public access to its data, which can be used as a valuable resource for diverse epidemiological investigations. The KCHS data for the years covered by this study were anonymized, and written informed consent was obtained from all participants before they participated in the study. Since there was no need to provide compensation or medical finding reports to participants, anonymized data were used in this study.

### Study Population and Data Collection

This study aimed to determine the sociodemographic factors associated with influenza and SARS-CoV-2 vaccination rates before and during the pandemic, respectively, and to identify vulnerable populations for vaccination. The data used in this study were from the KCHS conducted by the Korean Disease Control and Prevention Agency, which covers adults aged 19 years and older in South Korea, and the years analyzed are 2019, 2021, and 2022 [13]. Our study initially included 689,660 Korean adult population, aged 19 years or older. From this group, a total of 181,696 individuals were excluded due to missing data: 134,916 due to household income; 35,635 due to occupational categories; and 11,145 due to BMI. After applying these exclusion criteria, a nationally representative sample of 507,964 individuals was finalized for analysis. This sample was designed to reflect the diversity of the adult population in Korea (Figure S1 in Multimedia Appendix 1). Statistical analyses were performed by using weighting and sample clustering techniques to ensure robustness and representativeness of the study results. To further minimize the response bias, KCHS applied correction weights that adjusted for sex and age population structures based on the registered population. This methodological approach enhances the reliability and generalizability of the findings.

### Ascertainment of Vaccination

We aimed to comprehend the impact of age-specific vaccination policies and the pandemic on influenza and SARS-CoV-2 vaccination behaviors and to identify vulnerable groups. Given that the first case of COVID-19 in South Korea occurred on January 9, 2020, we designated the before-pandemic period as 2019 and the pandemic period as 2021 and 2022 [14]. The year 2020 was excluded due to the absence of SARS-CoV-2 vaccination information. Individuals were queried with targeted questions regarding their influenza and SARS-CoV-2 vaccination history, specifically asking, “Have you received an influenza vaccination in the past year?” and “Have you been vaccinated against COVID-19?”, respectively [8]. Based on the responses, if an individual had received at least 1 dose of each vaccine, they were defined as having received the vaccination for the respective vaccines. To investigate the influence of age-specific policies, we divided the cohorts into 2 groups: individuals aged 19‐64 years as the younger group, and individuals aged 65 years or older as the older group. Subsequently, we proceeded to compare SARS-CoV-2 vaccination to influenza vaccination before and during the pandemic within each cohort. In this study, the older group was defined as individuals aged 65 years or older according to South Korea’s vaccination policy and the World Health Organization’s age classification criteria [15].

### Definition of Covariates

This study used the Korean adult population based on KCHS as follows: age (19‐29, 30‐49, 50‐64, 65‐74, and ≥75 years), sex (male and female), region of residence (urban and rural) [16], education (elementary school or lower education, middle school, high school, and college or higher education), household income (lowest, second, third, and highest quartile), BMI group (underweight, normal weight, overweight, and obese), economic activity status, occupational categories (employer or self-employed, salaried, and unemployed), marital status, smoking status (nonsmoker, ex-smoker, and smoker), alcohol intake (nondrinker and drinker), hypertension, diabetes, sufficient physical activity, general health status (third and highest, second, and lowest quartile), depression, and unmet needs for health care services. BMI values were categorized into underweight (<18.5 kg/m^2^), normal weight (18.5‐22.9 kg/m^2^), overweight (23‐25 kg/m^2^), and obese (≥25.0 kg/m^2^) categories according to Asian-Pacific guidelines [17,18]. Sufficient physical activity was categorized into 2 groups according to the metabolic equivalent of task (MET) score by the World Health Organization physical activity guidelines: sufficient (≥600 MET minutes/week) and insufficient (<600 MET minutes/week) [13]. General health status was categorized into 4 quartiles by the EQ-5D-3L [19]. Depression was defined as a population with a score of 10 or higher on the Patient Health Questionnaire-9 [20].

### Statistical Analyses

To compare relevant risk factors affecting influenza and SARS-CoV-2 vaccination rates, we used a weighted multivariate logistic regression model with vaccination status as the dependent variable. Due to the nature of cross-sectional studies, it is difficult to directly calculate the frequency of disease occurrence at any given point in time, so using odds ratios can make the interpretation of results more intuitive when risk or incidence rates cannot be accurately estimated [21]. This model allows us to adjust for the representativeness of each participant, address potential collinearity between covariates, and assess the independent effect of each variable.

A variance inflation factor check revealed that most variance inflation factor values were 5 or less, indicating that the model does not have significant collinearity issues [22]. To estimate the interaction term for the 2 vaccinations, we stratified by age, using age 65 years as a cutoff point. We also calculated ratio of odds ratios (RORs) to identify groups that are particularly vulnerable to low vaccination rates and to assess interaction effects, further clarifying the complex relationships within the model. This approach improves the robustness and reliability of the results by maintaining the independence of the variables throughout the analysis.

Overall, this method, which used weighted multivariate logistic regression models, aimed to provide a comprehensive and robust assessment of the different demographic factors that influence influenza and SARS-CoV-2 vaccination rates [23,24]. Our model specifically aimed to identify vulnerable groups with low vaccination rates for each vaccine. These statistical methods have been effective in studying the association between demographic factors and vaccination outcomes across different age groups and periods, which can give us insight into the dynamics affecting vaccination programs and ultimately facilitate targeted interventions to improve public health outcomes. SAS software (version 9.4; SAS Institute) was used for statistical analysis, and a 2-sided *P*<.05 was considered statistically significant [25-27].

## Results

Table 1 shows the weighted baseline characteristics of the study population who participated in the KCHS in 2019, 2021, and 2022. Weighted values and their 95% CIs were presented to account for demographic disparities and ensure the representativeness of the Korean adult population. A comprehensive cohort of 507,964 Korean adults (mean age 55.20, SD 14.52 years; 95% CI 55.16‐55.24; male 48.16%; 95% CI 48.00‐48.32) was analyzed. Crude characteristics of baseline characteristics are shown in Table S1 in Multimedia Appendix 1.

**Table 1.** Weighted characteristics of Korean adults based on data obtained from the KCHS^a^ from 2019, 2021, and 2022 (N=507,964).

Variables	Total, weighted % (95% CI)	Before pandemic (2019)		During pandemic (2021‐2022)	
		Influenza vaccinated (n=99,978), weighted % (95% CI)	Influenza unvaccinated (n=77,467), weighted % (95% CI)	SARS-CoV-2 vaccinated (n=293,433), weighted % (95% CI)	SARS-CoV-2 unvaccinated (n=37,086), weighted % (95% CI)
**Age (years)**					
19‐29	16.00 (15.82-16.18)	2.65 (2.56-2.73)	8.11 (7.97-8.26)	12.83 (12.64-13.03)	4.02 (3.91-4.14)
30‐49	31.97 (31.72-32.22)	10.08 (9.91-10.25)	17.75 (17.54-17.96)	25.59 (25.33-25.85)	7.06 (6.90-7.21)
50‐64	28.05 (27.87-28.24)	13.66 (13.48-13.84)	16.23 (16.04-16.42)	26.21 (26.00-26.42)	1.54 (1.48-1.60)
65‐74	13.72 (13.57-13.86)	15.14 (14.95-15.33)	2.11 (2.04-2.18)	12.64 (12.48-12.80)	0.50 (0.47-0.53)
≥75	10.26 (10.14-10.39)	13.33 (13.15-13.51)	0.94 (0.90-0.99)	9.11 (8.97-9.24)	0.50 (0.47-0.53)
**Sex**					
Male	48.16 (48.00-48.32)	24.18 (23.99-24.37)	25.39 (25.19-25.60)	40.97 (40.77-41.16)	6.96 (6.82-7.10)
Female	51.84 (51.68-52.00)	30.67 (30.48-30.86)	19.75 (19.58-19.93)	45.41 (45.21-45.62)	6.66 (6.53-6.79)
**Region of residence**					
Urban	79.92 (79.65-80.20)	29.52 (29.25-29.79)	30.42 (30.14-30.70)	71.57 (71.27-71.88)	11.63 (11.41-11.84)
Rural	20.08 (19.80-20.35)	25.33 (25.10-25.56)	14.73 (14.46-14.99)	14.81 (14.58-15.04)	1.99 (1.92-2.07)
**Education**					
High school or lower education	50.07 (49.80-50.35)	40.81 (40.53-41.09)	22.36 (22.14-22.59)	42.81 (42.53-43.09)	5.12 (5.00-5.24)
College or higher education	49.93 (49.65-50.20)	14.05 (13.85-14.24)	22.78 (22.55-23.02)	43.58 (43.26-43.89)	8.50 (8.33-8.67)
**Household income**					
Lowest and second quartile income	35.59 (35.29-35.90)	31.21 (30.92-31.50)	13.73 (13.52-13.94)	29.73 (29.43-30.03)	4.33 (4.22-4.45)
Third and highest quartile income	64.41 (64.10-64.71)	23.64 (23.39-23.90)	31.42 (31.12-31.71)	56.65 (56.31-57.00)	9.29 (9.10-9.47)
**BMI^b^**					
Underweight or normal	44.32 (44.11-44.52)	18.54 (18.37-18.72)	16.84 (16.66-17.02)	39.07 (38.83-39.31)	6.71 (6.57-6.84)
Overweight or obese	55.68 (55.48-55.89)	36.31 (36.06-36.56)	28.31 (28.07-28.55)	47.31 (47.07-47.55)	6.91 (6.77-7.05)
**Economic activity status**					
Yes	59.83 (59.61-60.04)	29.58 (29.34-29.82)	32.82 (32.57-33.07)	51.35 (51.10-51.61)	8.06 (7.89-8.22)
No	40.17 (39.96-40.39)	25.28 (25.05-25.51)	12.33 (12.17-12.49)	35.03 (34.78-35.28)	5.56 (5.44-5.68)
**Occupational categories**					
Employer or self-employed	14.60 (14.45-14.74)	9.44 (9.30-9.59)	8.80 (8.65-8.95)	12.31 (12.16-12.46)	1.69 (1.63-1.75)
Salaried	45.22 (44.99-45.44)	20.08 (19.87-20.28)	23.97 (23.74-24.19)	39.04 (38.79-39.29)	6.37 (6.22-6.51)
Unemployed	40.19 (39.97-40.40)	25.33 (25.10-25.57)	12.38 (12.22-12.54)	35.03 (34.78-35.28)	5.56 (5.44-5.68)
**Marital status**					
Yes	60.47 (60.22-60.71)	38.08 (37.80-38.36)	27.79 (27.54-28.04)	53.04 (52.76-53.32)	6.54 (6.39-6.69)
No	39.53 (39.29-39.78)	16.77 (16.58-16.96)	17.35 (17.16-17.55)	33.34 (33.08-33.61)	7.08 (6.93-7.22)
**Smoking status**					
Nonsmoker	62.76 (62.57-62.94)	34.97 (34.75-35.20)	24.99 (24.77-25.21)	54.74 (54.50-54.98)	8.47 (8.31-8.63)
Ex-smoker	20.09 (19.94-20.25)	13.14 (12.98-13.29)	8.68 (8.54-8.81)	17.75 (17.58-17.92)	2.07 (1.99-2.14)
Smoker	17.15 (16.99-17.31)	6.75 (6.62-6.87)	11.48 (11.32-11.64)	13.90 (13.73-14.06)	3.08 (2.99-3.17)
**Alcohol intake**					
Nondrinker	49.45 (49.22-49.68)	31.80 (31.55-32.04)	16.99 (16.80-17.19)	42.95 (42.69-43.20)	6.61 (6.47-6.74)
Drinker	50.55 (50.32-50.78)	23.06 (22.84-23.28)	28.15 (27.91-28.40)	43.43 (43.16-43.70)	7.01 (6.86-7.16)
**Hypertension**					
Yes	23.72 (23.54-23.90)	22.21 (21.99-22.43)	7.22 (7.09-7.35)	21.33 (21.14-21.52)	1.46 (1.40-1.51)
No	76.28 (76.10-76.46)	32.65 (32.41-32.89)	37.92 (37.65-38.19)	65.05 (64.79-65.31)	12.16 (11.95-12.37)
**Diabetes**					
Yes	10.49 (10.37-10.61)	9.07 (8.93-9.22)	2.95 (2.87-3.03)	9.52 (9.39-9.65)	0.72 (0.68-0.76)
No	89.51 (89.39-89.63)	45.78 (45.51-46.05)	42.19 (41.91-42.47)	76.87 (76.63-77.10)	12.90 (12.69-13.11)
**Sufficient physical activity**					
Yes	31.86 (31.64-32.08)	18.22 (18.01-18.42)	17.53 (17.33-17.74)	27.19 (26.94-27.43)	4.03 (3.93-4.14)
No	68.14 (67.92-68.36)	36.64 (36.38-36.90)	27.61 (27.37-27.85)	59.19 (58.93-59.46)	9.58 (9.41-9.76)
**General health status**					
Third and highest quartile	65.37 (65.14-65.60)	29.51 (29.27-29.76)	32.78 (32.51-33.05)	56.45 (56.17-56.73)	9.42 (9.24-9.60)
Second quartile	12.99 (12.84-13.13)	7.64 (7.51-7.76)	5.35 (5.23-5.46)	11.56 (11.41-11.72)	1.42 (1.36-1.48)
Lowest quartile	21.65 (21.46-21.83)	17.70 (17.50-17.90)	7.02 (6.90-7.15)	18.36 (18.17-18.56)	2.78 (2.69-2.86)
**Depression**					
Yes	3.78 (3.70-3.86)	1.88 (1.82-1.95)	1.19 (1.13-1.24)	3.22 (3.14-3.31)	0.68 (0.64-0.72)
No	96.22 (96.14-96.30)	52.97 (52.69-53.25)	43.96 (43.68-44.24)	83.16 (82.93-83.39)	12.94 (12.73-13.15)
**Unmet needs for health care services**					
Insufficient	4.79 (4.69-4.88)	2.66 (2.58-2.74)	2.75 (2.67-2.83)	3.88 (3.78-3.97)	0.80 (0.76-0.85)
Sufficient	95.21 (95.12-95.31)	52.19 (51.91-52.47)	42.39 (42.12-42.67)	82.50 (82.27-82.73)	12.81 (12.60-13.02)

a KCHS: Korea Community Health Service.

b According to the Asian-Pacific guidelines, the BMI is divided into 4 groups: underweight (<18.5 kg/m2), normal (18.5-22.9 kg/m2), overweight (23.0-24.9 kg/m2), and obese (≥25.0 kg/m2).

**Figure 1.**
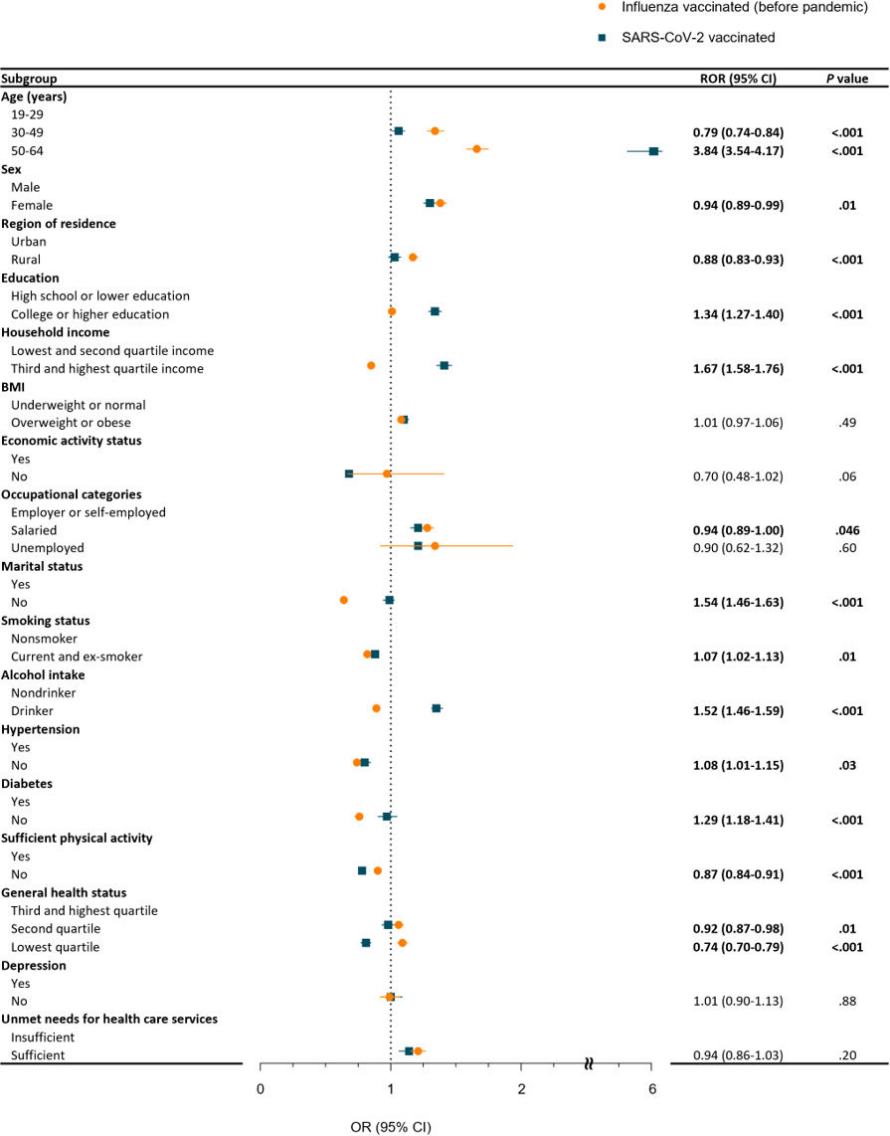
The difference in SARS-CoV-2 vaccination compared to influenza vaccination before the pandemic among individuals aged 19-64 years (weighted and adjusted OR, 95% CI). Numbers in bold format indicate a significant difference (*P*<.05). OR: odds ratio; ROR: ratio of odds ratio.

Figures1  2 describe the association of sociodemographic factors with vaccine acceptance among the younger cohort (19‐64 years), while Figures3  4 highlight the older cohort (65 years or older). Figure 1 and Table S2 in Multimedia Appendix 1 illustrate the trends in the association concerning the acceptance of influenza vaccination before the pandemic and SARS-CoV-2 vaccination during the pandemic within the younger demographic cohort. Factors exhibiting a higher association with SARS-CoV-2 vaccination, in contrast to influenza vaccination, were older age (ROR 3.84; 95% CI 3.54‐4.17), higher education level (ROR 1.34; 95% CI 1.27‐1.40), higher income (ROR 1.67; 95% CI 1.58‐1.76), unmarried status (ROR 1.54; 95% CI 1.46‐1.63), current or ex-smoking status (ROR 1.07; 95% CI 1.02‐1.13), current alcohol intake (ROR 1.52; 95% CI 1.46‐1.59), absence of hypertension (ROR 1.08; 95% CI 1.01‐1.15), and absence of diabetes (ROR 1.29; 95% CI 1.18‐1.41). Conversely, factors associated with SARS-CoV-2 vaccination hesitancy compared to influenza vaccination included female sex (ROR 0.94; 95% CI 0.89‐0.99), rural residence (ROR 0.88; 95% CI 0.83‐0.93), insufficient physical activity (ROR 0.87; 95% CI 0.84‐0.91), and lower general health status (ROR 0.74; 95% CI 0.70‐0.79).

**Figure 2.**
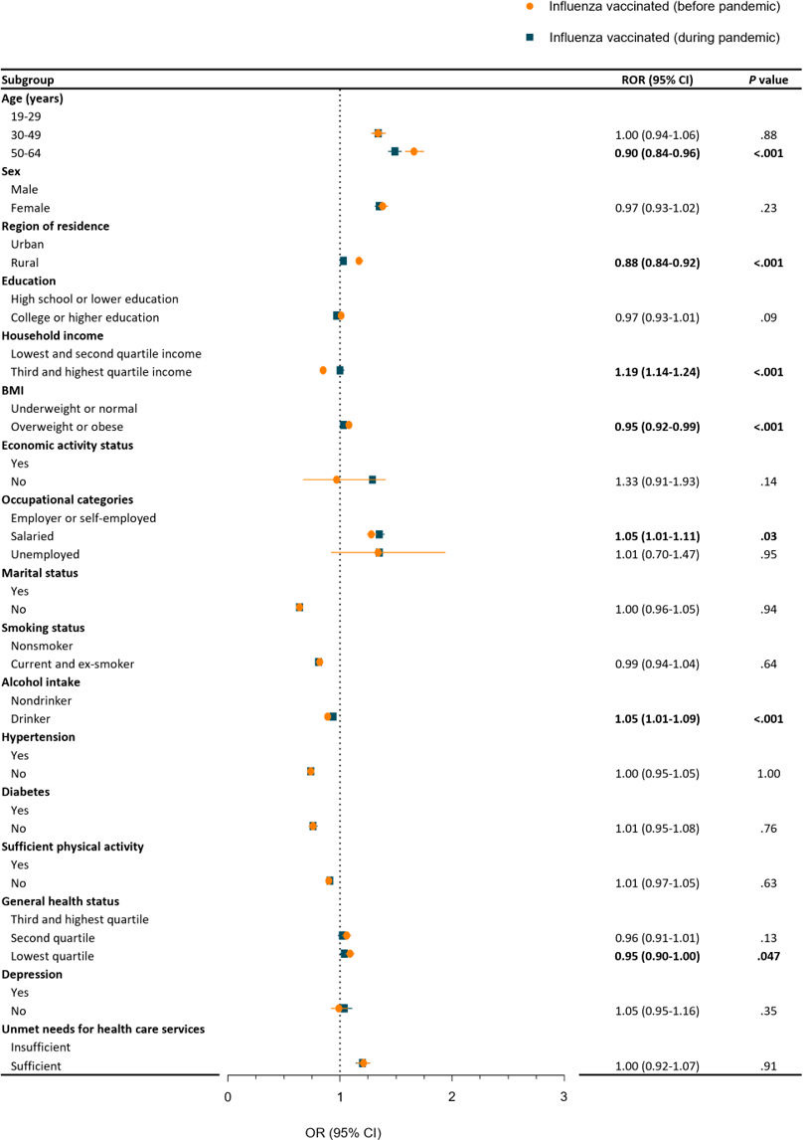
The difference in influenza vaccination before the pandemic compared to during the pandemic among individuals aged 19-64 years (weighted and adjusted OR, 95% CI]). Numbers in bold format indicate a significant difference (*P*<.05). OR: odds ratio; ROR: ratio of odds ratio.

**Figure 3.**
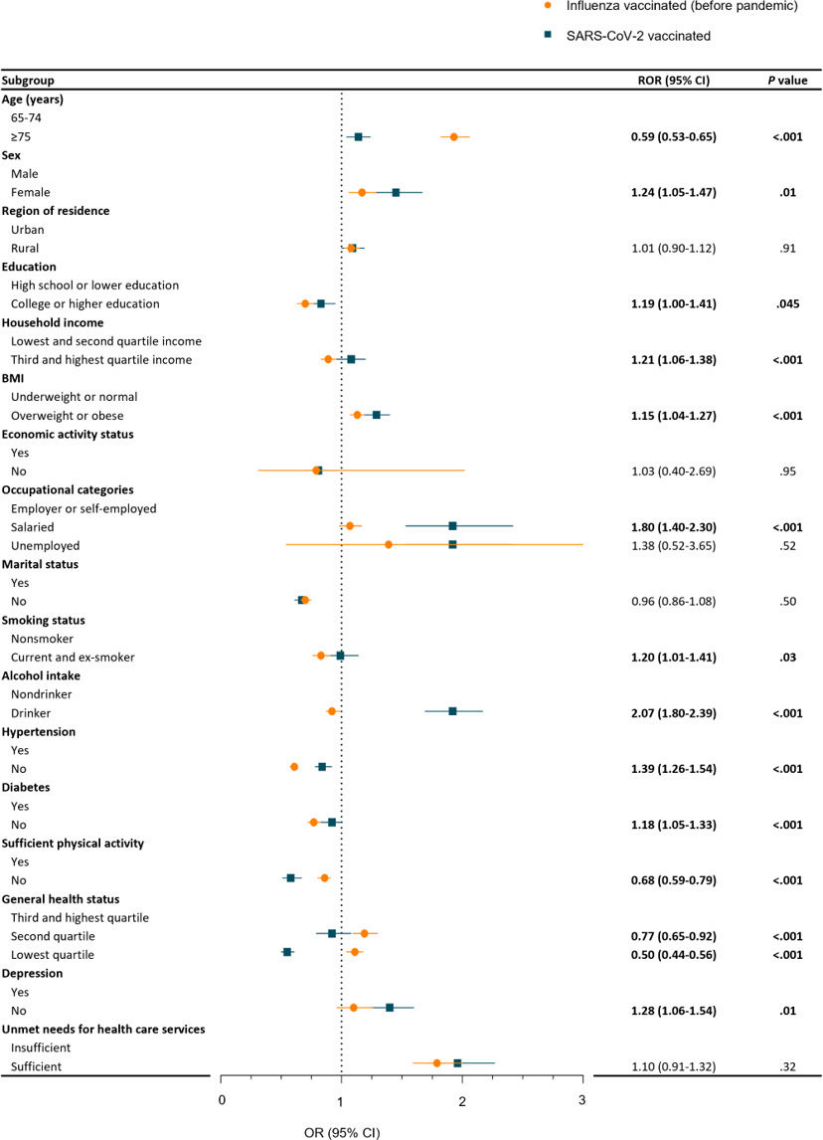
The difference in SARS-CoV-2 vaccination compared to influenza vaccination before the pandemic among individuals aged ≥65 years (weighted and adjusted OR, 95% CI). Numbers in bold format indicate a significant difference (*P*<.05). OR: odds ratio; ROR: ratio of odds ratio.

**Figure 4.**
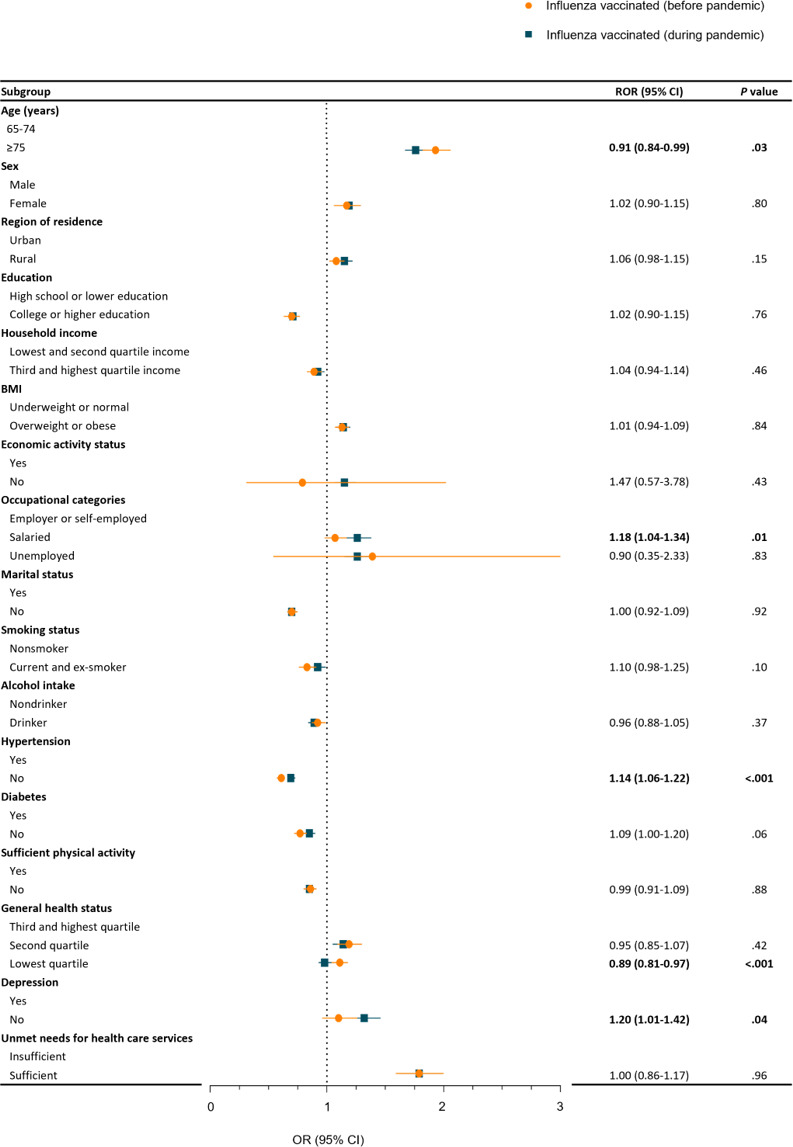
The difference in influenza vaccination before the pandemic compared to during the pandemic among individuals aged ≥65 years (weighted and adjusted OR, 95% CI). Numbers in bold format indicate a significant difference (*P*<.05). OR: odds ratio; ROR: ratio of odds ratio.

Figure 2 and Table S3 in Multimedia Appendix 1 depict the association with influenza vaccine acceptance within the younger cohort before and during the pandemic. Factors associated with higher influenza vaccination during the pandemic, compared to before the pandemic, include higher income (ROR 1.19; 95% CI 1.14‐1.24) and salaried occupations (ROR 1.05; 95% CI 1.01‐1.11). Conversely, factors exhibiting lower association with influenza vaccination during the pandemic as opposed to before, included age (ROR 0.90; 95% CI 0.84‐0.96), rural residence (ROR 0.88; 95% CI 0.84‐0.92), and overweight or obese (ROR 0.95; 95% CI 0.92‐0.99). Among the investigated variables, no significant differences in association were observed for other factors.

Figure 3 and Table S4 in Multimedia Appendix 1 represent the association of demographic factors with influenza vaccination before the pandemic and SARS-CoV-2 vaccination within the older cohort. Factors demonstrating a higher association with SARS-CoV-2 vaccination compared to influenza vaccination were female sex (ROR 1.24; 95% CI 1.05‐1.47), higher education level (ROR 1.19; 95% CI 1.00‐1.41), higher income (ROR 1.21; 95% CI 1.06‐1.38), overweight or obese (ROR 1.15; 95% CI 1.04‐1.27), salaried occupations (ROR 1.80; 95% CI 1.40‐2.30), current or ex-smoking status (ROR 1.20; 95% CI 1.01‐1.41), current alcohol consumption (ROR 2.07; 95% CI 1.80‐2.39), absence of hypertension (ROR 1.39; 95% CI 1.26‐1.54), absence of diabetes (ROR 1.18; 95% CI 1.05‐1.33), and absence of depression (ROR 1.28; 95% CI 1.06‐1.54). Conversely, factors associated with lower vaccination for COVID-19 compared to influenza included older age (ROR 0.59; 95% CI 0.53‐0.65), insufficient physical activity (ROR 0.68; 95% CI 0.59‐1.33), and lower general health status (ROR 0.50; 95% CI 0.44‐0.56).

Figure 4 and Table S5 in Multimedia Appendix 1 delineate the association of demographic factors with influenza vaccination before and during the pandemic within the older cohort. Salaried occupations (ROR 1.18; 95% CI 1.04‐1.34), absence of hypertension (ROR 1.14; 95% CI 1.06‐1.22), and absence of depression (ROR 1.20; 95% CI 1.01‐1.42) showed increasing tendency for vaccination during the pandemic. Conversely, older age (ROR 0.91; 95% CI 0.84‐0.99) and lower general health status (ROR 0.89; 95% CI 0.81‐0.97) showed a decreasing tendency toward vaccination during the pandemic compared to before the pandemic.

Tables S6 and S7 in Multimedia Appendix 1 illustrate the association between various demographic factors and the uptake of influenza and SARS-CoV-2 vaccines among age groups 19‐64 years and those older than 65 years during the pandemic. Furthermore, Table S8 in Multimedia Appendix 1 details the associations of these risk factors with the uptake of influenza vaccines both before the pandemic and during the pandemic, and with SARS-CoV-2 vaccines, according to the KCHS data.

## Discussion

### Key Findings

In this study, we present a large-scale examination contrasting risk factors linked to influenza and SARS-CoV-2 vaccination within a nationally representative South Korean cohort (N=507,964). The results of our study emphasize that factors indicating higher socioeconomic status (SES), such as higher education, higher income, former or current smoking habits, and current alcohol consumption, were associated with increased acceptance of SARS-CoV-2 vaccination. Furthermore, for influenza vaccine acceptance during the pandemic, disparities were observed based on age, necessitating a precise age-specific vaccination strategy to enhance overall vaccine acceptance. While the older cohort indicated that low general health status was associated with low vaccination rates, in the younger cohort, individuals with lower income tended to be associated with nonvaccination.

### Comparison of Previous Studies

The influence of the COVID-19 pandemic on the acceptance of SARS-CoV-2 vaccines has been demonstrated in several studies [28-31]. Our study has a substantial sample size, surpassing the sample sizes of comparable research in other countries: United States (n=2978) [28], China (n=34,291) [29], Canada (n=2522) [30], Pakistan (n=384) [31], and England (n=1058) [32]. Consistent with studies conducted in the United States, Canada, and Pakistan, our findings emphasize the association of higher income and education levels with increased vaccine acceptance. Furthermore, our studies further developed into comparing SARS-CoV-2 vaccination behaviors with influenza vaccination. Previous research indicates that free vaccination policies for older populations significantly increase vaccination prevalence [33]. Consistent with these findings, our results show that older populations, who receive free vaccinations, did not exhibit a significant association between income levels and vaccination prevalence, suggesting that removing financial barriers can effectively increase vaccination prevalence. In contrast, our study found that the younger cohort, who are required to pay for influenza vaccinations, showed a higher association between lower income levels and vaccine hesitancy. This shows the impact of financial constraints on vaccine acceptance among younger individuals. Comparing these results with studies from other countries that have implemented age-specific vaccination policies further supports the importance of financial accessibility in increasing vaccination coverage. We also uniquely explored the influence of age-specific vaccination policies during the COVID-19 pandemic.

### Plausible Underlying Mechanisms

Individuals with higher income, higher education, current or former smoking habits, and alcohol consumption showed increased SARS-CoV-2 vaccine acceptance compared to influenza vaccine acceptance before the pandemic [34]. This observation may be attributed to the SES characteristic of these cohorts [35,36]. Higher SES is often associated with better access to health information and resources, leading to increased health-seeking behavior [37]. The heightened acceptance of the SARS-CoV-2 vaccine among these groups may also be driven by the desire to maintain their SES and adhere to health-protective behaviors during the pandemic [9]. Due to government directives on social distancing, to continue SES, vaccination was nearly necessitated. Moreover, individuals with these factors exhibit a greater tendency for social interaction, thereby fostering an environment favorable to spreading health-related information [38,39].

The same factors exhibited a correlation with SARS-CoV-2 vaccination uptake among the older cohort but had a faint effect compared to the younger cohort. This could potentially be attributed to the inherently diminished levels of social participation observed within the older population [40].

For influenza vaccination during the pandemic in the younger demographic, a significant association was observed between higher income levels and influenza vaccine acceptance [41]. This correlation may be elucidated by the enhanced financial capacity of individuals with higher incomes to afford the cost of vaccines [42]. Among the older demographic, an association was identified between lower general health status and influenza vaccine hesitancy during the pandemic. Presumably, in this context, accessibility to medical services and mobility of health care services emerged as a more influential determinant, as individuals aged 65 years and older were free of charge for all types of vaccination [43]. To address these disparities, we examined how specific SES factors create barriers to vaccination. Lower income limits financial resources for vaccines, leading to lower acceptance rates among the younger population. Additionally, lower educational attainment may reduce health literacy, resulting in increased vaccine hesitancy. For older adults, limited mobility and reduced access to health care services significantly impact vaccination uptake despite the availability of free vaccines.

### Limitations

While this study revealed associations between sociodemographic factors and vaccination uptake during the pandemic, it has some limitations due to the inherent characteristics of the KCHS database. First, the presence of unmeasured variables, such as history of disease or allergies, may have influenced vaccination acceptance, introducing a potential source of bias [44]. Second, our analysis exclusively encompasses data from the Korean adult population; therefore, the findings may not be generalized in other countries, caused by cultural and regional differences. Third, the stratification of the population based on self-reported data, such as alcohol consumption and sufficient physical activity, introduces social desirability bias and recall bias [45]. Fourth, the KCHS, being conducted face-to-face, could not be carried out in 2020 due to the onset of the COVID-19 pandemic. Therefore, we defined the pandemic period in our study as 2021‐2022. For before the pandemic, we chose the year 2019, which was significantly impacted by the pandemic, as data for many sociodemographic factors we intended to analyze were missing in the years prior. This limitation might have resulted in the omission of relevant sociodemographic data from earlier years that were not collected. Fifth, our study may not include all the potential factors influencing vaccination behavior, such as psychological factors and individual health conditions. Finally, the cross-sectional nature of this study limits our ability to distinguish between correlation and causation between variables; therefore, it is important to consider the nature of the study when interpreting the associations observed in our result.

Despite the acknowledged limitations, this study has several strengths and contributes valuable insights to public health strategies. First, our study benefits from a substantial sample size (N=507,964), providing nationwide representative data of the Korean adult population, and weighted adjustments have been applied for enhanced accuracy and representativeness. Second, to our knowledge, this is the first study to compare the vaccination behavior of nonpandemic disease (influenza) and pandemic disease (COVID-19) within the context of age-specific policies. This approach enhances our understanding of how different age groups respond to vaccination initiatives during varying public health circumstances. Third, we conducted a thorough examination of various subgroups, providing a comprehensive analysis of distinct characteristics under varied conditions and diseases.

### Policy Implications

Our study presents the influence of sociodemographic factors in shaping vaccination behaviors, revealing the necessity of careful consideration in public health strategies and interventions [46]. It is crucial to address the trend observed in both younger and older populations, the association between factors possibly indicating low SES and SARS-CoV-2 vaccine hesitancy. Barriers such as limited financial resources, low educational levels, and limited access to health care services may have an impact on vaccination acceptance. Therefore, to overcome these barriers on individuals with low SES, who are more strongly associated with vaccine hesitancy, developing targeted vaccination strategies such as educational campaigns addressing common concerns, incentives to encourage vaccination, and community-based vaccination clinics is essential to reduce access barriers. Developing targeted vaccination campaigns tailored to communities with low social participation rates is needed.

Within the older cohort, where vaccines are provided free of charge, lower general health status was associated with lower influenza vaccination. However, in the younger cohort responsible for vaccine expenses, lower income was associated with vaccine hesitancy. To address these disparities and enhance public health, additional age-specific policies are required. For the older population, governments should support health care services to improve mobility, particularly in regions with an aging population and limited health infrastructure. Establishing mobile vaccination units that visit communities with a high proportion of older residents can significantly improve their vaccination [47,48]. Additionally, campaigns targeting older populations in areas with limited health infrastructure can help change perceptions and increase vaccine acceptance. Furthermore, to counter vaccine hesitancy among younger populations with lower income, it is advisable to explore policies that offer free vaccination or financial support for this cohort. These considerations aim to amplify public health, particularly in the face of potential future pandemics [49].

### Conclusions

This study addresses age-specific vaccination policies, particularly in the context of the COVID-19 pandemic. This large-scale representative research aimed to improve age-specific policies by examining the association between sociodemographic factors and vaccine acceptance. For SARS-CoV-2 vaccination, a free vaccination regardless of age, factors potentially indicating high SES, such as higher income, higher education, current or former smoking habits, and alcohol consumption, showed higher vaccine acceptance. This association was more pronounced within the younger cohort. For influenza vaccination, a vaccine with age-specific costs, the older cohort showed vaccination hesitancy associated with lower mobility of health care, while the younger cohort showed hesitancy associated with lower income. Addressing these disparities and developing targeted vaccination strategies to enhance public vaccine acceptance are crucial for preventing future pandemics. Further research to investigate the lasting impact on vaccination behaviors after the pandemic is required.

## Supplementary material

10.2196/56989 Multimedia Appendix 1 Association between sociodemographic factors and vaccine acceptance for influenza and SARS-CoV-2 in South Korea based on data obtained from the Korean Community Health Survey from 2019, 2021, and 2022.

## Abbreviations

KCHS: Korean Community Health Survey
MET: metabolic equivalent of task
ROR: ratio of odds ratio
SES: socioeconomic status
