# Supplementary material for: Association Between Sociodemographic Factors and Vaccine Acceptance for Influenza and SARS-CoV-2 in South Korea: Nationwide Cross-Sectional Study
Source: JMIR Public Health Surveill. 2024 Nov 1;10:e56989. doi: 10.2196/56989 (PMC11615830; doi:10.2196/56989)
Supplement: Multimedia Appendix 1 [file publichealth-v10-e56989-s001.docx]

**Figure S1.** Study population


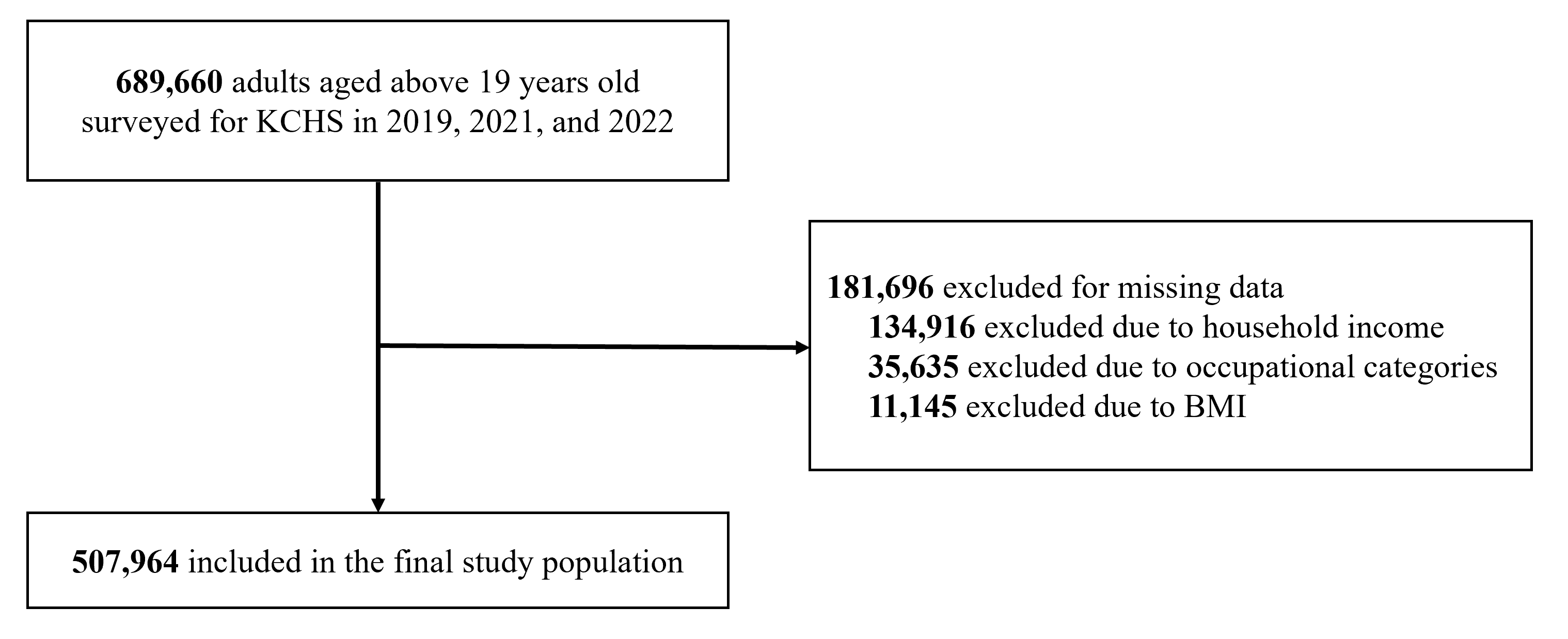


**Table S1.** Crude characteristics of Korean adults based on data obtained from the KCHS from 2019, 2021, and 2022 (N=507,964)

| Variables | Total | Before pandemic (2019) | | During pandemic (2021–2022) | |  |
| --- | --- | --- | --- | --- | --- | --- |
|  |  | Influenza vaccinated | Influenza unvaccinated | SARS–CoV–2 vaccinated | SARS–CoV–2 unvaccinated |  |
|  |  |  |  |  |  |  |
| Overall, n | 507,964 | 99,978 | 77,467 | 293,433 | 37,086 |  |
| Age, n (%) |  |  |  |  |  |  |
| 19–29 | 54,678 (10.76) | 4,544 (2.56) | 13,921 (7.85) | 27,522 (8.33) | 8,691 (2.63) |  |
| 30–49 | 131,260 (25.84) | 17,154 (9.67) | 29,545 (16.65) | 66,395 (20.09) | 18,166 (5.50) |  |
| 50–64 | 148,953 (29.32) | 24,288 (13.69) | 28,247 (15.92) | 90,989 (27.53) | 5,429 (1.64) |  |
| 65–74 | 92,348 (18.18) | 27,613 (15.56) | 3,828 (2.16) | 58,699 (17.76) | 2,208 (0.67) |  |
| ≥75 | 80,725 (15.89) | 26,379 (14.87) | 1,926 (1.09) | 49,828 (15.08) | 2,592 (0.78) |  |
| Sex, n (%) |  |  |  |  |  |  |
| Male | 225,980 (44.49) | 39,633 (22.34) | 39,230 (22.11) | 129,365 (39.14) | 17,752 (5.37) |  |
| Female | 281,984 (55.51) | 60,345 (34.01) | 38,237 (21.55) | 164,068 (49.64) | 19,334 (5.85) |  |
| Region of residence, n (%) |  |  |  |  |  |  |
| Urban | 306,226 (60.29) | 52,898 (29.81) | 52,187 (29.41) | 175,605 (53.13) | 25,536 (7.73) |  |
| Rural | 201,738 (39.72) | 47,080 (26.53) | 25,280 (14.25) | 117,828 (35.65) | 11,550 (3.49) |  |
| Education, n (%) |  |  |  |  |  |  |
| High school or lower education | 314,074 (61.83) | 75,887 (42.77) | 39,100 (22.04) | 182,302 (55.16) | 16,785 (5.08) |  |
| College or higher education | 193,890 (38.17) | 24,091 (13.58) | 38,367 (21.62) | 111,131 (33.62) | 20,301 (6.14) |  |
| Household income, n (%) |  |  |  |  |  |  |
| Lowest and second quartile income | 232,114 (45.70) | 58,556 (33.00) | 24,118 (13.59) | 135,038 (40.86) | 14,402 (4.36) |  |
| Third and highest quartile income | 275,850 (54.31) | 41,422 (23.34) | 53,349 (30.07) | 158,395 (47.92) | 22,684 (6.86) |  |
| BMI, n (%) |  |  |  |  |  |  |
| Underweight or normal | 224,201 (44.14) | 39,234 (22.11) | 33,831 (19.07) | 132,614 (40.12) | 18,522 (5.60) |  |
| Overweight or obese | 283,763 (55.86) | 60,744 (34.23) | 43,636 (24.59) | 160,819 (48.66) | 18,564 (5.62) |  |
| Economic activity status, n (%) |  |  |  |  |  |  |
| Yes | 295,672 (58.21) | 52,225 (29.43) | 55,109 (31.06) | 167,539 (50.69) | 20,799 (6.29) |  |
| No | 212,292 (41.79) | 47,753 (26.91) | 22,358 (12.60) | 125,894 (38.09) | 16,287 (4.93) |  |
| Occupational categories, n (%) |  |  |  |  |  |  |
| Employer or self–employed | 85,972 (16.92) | 16,512 (9.31) | 14,521 (8.18) | 49,914 (15.10) | 5,025 (1.52) |  |
| Salaried | 209,505 (41.24) | 35,606 (20.07) | 40,500 (22.82) | 117,625 (35.59) | 15,774 (4.77) |  |
| Unemployed | 212,487 (41.83) | 47,860 (26.97) | 22,446 (12.65) | 125,894 (38.09) | 16,287 (4.93) |  |
| Marital status, n (%) |  |  |  |  |  |  |
| Yes | 319,820 (62.96) | 67,998 (38.32) | 47,594 (26.82) | 185,811 (56.22) | 18,417 (5.57) |  |
| No | 188,144 (37.04) | 31,980 (18.02) | 29,873 (16.84) | 107,622 (32.56) | 18,669 (5.65) |  |
| Smoking status, n (%) |  |  |  |  |  |  |
| Non–smoker | 325,298 (64.04) | 66,821 (37.66) | 45,647 (25.72) | 189,534 (57.34) | 23,296 (7.05) |  |
| Ex–smoker | 101,858 (20.05) | 21,962 (12.38) | 13,670 (7.70) | 60,626 (18.34) | 5,600 (1.69) |  |
| Smoker | 80,808 (15.91) | 11,195 (6.31) | 18,150 (10.23) | 43,273 (13.09) | 8,190 (2.48) |  |
| Alcohol intake, n (%) |  |  |  |  |  |  |
| Non–drinker | 274,460 (54.03) | 60,109 (33.87) | 30,377 (17.12) | 164,496 (49.77) | 19,478 (5.89) |  |
| Drinker | 233,504 (45.97) | 39,869 (22.47) | 47,090 (26.54) | 128,937 (39.01) | 17,608 (5.33) |  |
| Hypertension, n (%) |  |  |  |  |  |  |
| Yes | 150,260 (29.58) | 40,150 (22.63) | 11,803 (6.65) | 92,965 (28.13) | 5,342 (1.62) |  |
| No | 357,704 (70.42) | 59,828 (33.72) | 65,664 (37.01) | 200,468 (60.65) | 31,744 (9.60) |  |
| Diabetes, n (%) |  |  |  |  |  |  |
| Yes | 64,469 (12.69) | 16,198 (9.13) | 4,874 (2.75) | 40,752 (12.33) | 2,645 (0.80) |  |
| No | 443,495 (87.31) | 83,780 (47.21) | 72,593 (40.91) | 252,681 (76.45) | 34,441 (10.42) |  |
| Sufficient physical activity, n (%) |  |  |  |  |  |  |
| Yes | 158,688 (31.24) | 32,198 (18.15) | 29,351 (16.54) | 86,784 (26.26) | 10,355 (3.13) |  |
| No | 349,276 (68.76) | 67,780 (38.20) | 48,116 (27.12) | 206,649 (62.52) | 26,731 (8.09) |  |
| General health status, n (%) |  |  |  |  |  |  |
| Third and highest quartile | 308,823 (60.80) | 52,295 (29.47) | 55,718 (31.40) | 176,522 (53.41) | 24,288 (7.35) |  |
| Second quartile | 67,994 (13.39) | 13,948 (7.86) | 9,208 (5.19) | 40,907 (12.38) | 3,931 (1.19) |  |
| Lowest quartile | 131,147 (25.82) | 33,735 (19.01) | 12,541 (7.07) | 76,004 (23.00) | 8,867 (2.68) |  |
| Depression, n (%) |  |  |  |  |  |  |
| Yes | 18,705 (3.68) | 3,615 (2.04) | 2,117 (1.19) | 10,949 (3.31) | 2,024 (0.61) |  |
| No | 489,259 (96.32) | 96,363 (54.31) | 75,350 (42.46) | 282,484 (85.47) | 35,062 (10.61) |  |
| Unmet needs for healthcare services, n (%) |  |  |  |  |  |  |
| Insufficient | 26,043 (5.13) | 4,976 (2.80) | 4,787 (2.70) | 13,915 (4.21) | 2,365 (0.72) |  |
| Sufficient | 481,921 (94.87) | 95,002 (53.54) | 72,680 (40.96) | 279,518 (84.57) | 34,721 (10.51) |  |

Abbreviations: BMI, body mass index; CI, confidence interval; KCHS, Korea Community Health Service.

* According to the Asian–Pacific guidelines, the BMI is divided into four groups: underweight (<18.5 kg/m^2^), normal (18.5–22.9 kg/m^2^), overweight (23.0–24.9 kg/m^2^), and obese (≥25.0 kg/m^2^).

**Table S2.** Adjusted and weighted odds ratios for influenza before pandemic and SARS–CoV–2 vaccination in individuals aged 19–64

| Variables | | Influenza vaccinated (before pandemic) | | SARS–CoV–2 vaccinated | | Ratio of ORs (95% CI), SARS–CoV–2 vaccinated compared to influenza vaccinated (before pandemic) | |
| --- | --- | --- | --- | --- | --- | --- | --- |
|  |  | Weighted OR (95% CI) | P–value | Weighted OR (95% CI) | P–value | Ratio of ORs (95% CI) | P–value |
| 19–64 years old | | | | | | | |
| Age | 19–29 | 1.00 (ref) |  | 1.00 (ref) |  |  |  |
|  | 30–49 | **1.34 (1.28 to 1.41)** | **<.001** | **1.06 (1.01 to 1.11)** | **0.015** | **0.79 (0.74 to 0.84)** | **<.001** |
|  | 50–64 | **1.66 (1.58 to 1.75)** | **<.001** | **6.37 (5.97 to 6.80)** | **<.001** | **3.84 (3.54 to 4.17)** | **<.001** |
| Sex | Male | 1.00 (ref) |  | 1.00 (ref) |  |  |  |
|  | Female | **1.38 (1.34 to 1.43)** | **<.001** | **1.30 (1.25 to 1.35)** | **<.001** | **0.94 (0.89 to 0.99)** | **0.013** |
| Region of residence | Urban | 1.00 (ref) |  | 1.00 (ref) |  |  |  |
|  | Rural | **1.17 (1.14 to 1.21)** | **<.001** | 1.03 (0.98 to 1.08) | 0.198 | **0.88 (0.83 to 0.93)** | **<.001** |
| Education | High school or lower education | 1.00 (ref) |  | 1.00 (ref) |  |  |  |
|  | College or higher education | 1.01 (0.98 to 1.04) | 0.717 | **1.34 (1.29 to 1.39)** | **<.001** | **1.34 (1.27 to 1.40)** | **<.001** |
| Household income | Lowest and second quartile income | 1.00 (ref) |  | 1.00 (ref) |  |  |  |
|  | Third and highest quartile income | **0.85 (0.82 to 0.87)** | **<.001** | **1.41 (1.35 to 1.47)** | **<.001** | **1.67 (1.58 to 1.76)** | **<.001** |
| BMI | Underweight or normal | 1.00 (ref) |  | 1.00 (ref) |  |  |  |
|  | Overweight or obese | **1.08 (1.06 to 1.11)** | **<.001** | **1.10 (1.06 to 1.14)** | **<.001** | 1.01 (0.97 to 1.06) | 0.492 |
| Economic activity status | Yes | 1.00 (ref) |  | 1.00 (ref) |  |  |  |
|  | No | 0.97 (0.67 to 1.41) | 0.878 | **0.68 (0.66 to 0.71)** | **<.001** | 0.70 (0.48 to 1.02) | 0.061 |
| Occupational categories | Employer or self–employed | 1.00 (ref) |  | 1.00 (ref) |  |  |  |
|  | Salaried | **1.28 (1.24 to 1.33)** | **<.001** | **1.21 (1.15 to 1.27)** | **<.001** | 0.94 (0.89 to 1.00) | 0.046 |
|  | Unemployed | 1.34 (0.92 to 1.94) | 0.127 | **1.21 (1.15 to 1.27)** | **<.001** | 0.90 (0.62 to 1.32) | 0.597 |
| Marital status | Yes | 1.00 (ref) |  | 1.00 (ref) |  |  |  |
|  | No | **0.64 (0.62 to 0.66)** | **<.001** | 0.99 (0.94 to 1.03) | 0.519 | **1.54 (1.46 to 1.63)** | **<.001** |
| Smoking status | Non–smoker | 1.00 (ref) |  | 1.00 (ref) |  |  |  |
|  | Current and ex–smoker | **0.82 (0.79 to 0.85)** | **<.001** | **0.88 (0.84 to 0.91)** | **<.001** | **1.07 (1.02 to 1.13)** | **0.009** |
| Alcohol intake | Non–drinker | 1.00 (ref) |  | 1.00 (ref) |  |  |  |
|  | Drinker | **0.89 (0.87 to 0.91)** | **<.001** | **1.35 (1.31 to 1.40)** | **<.001** | **1.52 (1.46 to 1.59)** | **<.001** |
| Hypertension | Yes | 1.00 (ref) |  | 1.00 (ref) |  |  |  |
|  | No | **0.74 (0.72 to 0.77)** | **<.001** | **0.80 (0.76 to 0.85)** | **<.001** | **1.08 (1.01 to 1.15)** | **0.029** |
| Diabetes | Yes | 1.00 (ref) |  | 1.00 (ref) |  |  |  |
|  | No | **0.76 (0.72 to 0.79)** | **<.001** | 0.97 (0.90 to 1.05) | 0.505 | **1.29 (1.18 to 1.41)** | **<.001** |
| Sufficient physical activity | Yes | 1.00 (ref) |  | 1.00 (ref) |  |  |  |
|  | No | **0.90 (0.88 to 0.92)** | **<.001** | **0.78 (0.76 to 0.81)** | **<.001** | **0.87 (0.84 to 0.91)** | **<.001** |
| General health status | Third and highest quartile | 1.00 (ref) |  | 1.00 (ref) |  |  |  |
|  | Second quartile | **1.06 (1.02 to 1.10)** | **0.003** | 0.98 (0.93 to 1.03) | 0.348 | **0.92 (0.87 to 0.98)** | **0.014** |
|  | Lowest quartile | **1.09 (1.05 to 1.13)** | **<.001** | **0.81 (0.77 to 0.85)** | **<.001** | **0.74 (0.70 to 0.79)** | **<.001** |
| Depression | Yes | 1.00 (ref) |  | 1.00 (ref) |  |  |  |
|  | No | 0.99 (0.92 to 1.07) | 0.846 | 1.00 (0.92 to 1.09) | 0.986 | 1.01 (0.90 to 1.13) | 0.878 |
| Unmet needs for healthcare services | Insufficient | 1.00 (ref) |  | 1.00 (ref) |  |  |  |
|  | Sufficient | **1.21 (1.15 to 1.27)** | **<.001** | **1.14 (1.06 to 1.22)** | **<.001** | 0.94 (0.86 to 1.03) | 0.196 |

Abbreviations: BMI, body mass index; CI, confidence interval; OR, odds ratio.

The multivariable logistic regression was adjusted by age (19–29, 30–49, 50–64, 65–74, and ≥75 years), sex, region of residence (urban and rural), education (elementary school or lower education, middle school, high school, and college or higher education), household income (lowest, second, third, and highest quartile), BMI (underweight, normal weight, overweight, and obese), economic activity status, occupational categories (employer or self–employed, salaried, and unemployed), marital status, smoking status (non–smoker, ex–smoker, and smoker), alcohol intake (non–drinker, drinker), hypertension, diabetes, sufficient physical activity, general health status (third and highest, second, and lowest quartile), depression, and unmet needs for healthcare services (insufficient and sufficient).

The numbers in bold indicate significant differences (p<0.05).

**Table S3.** Adjusted and weighted odds ratios for influenza vaccination before and during the pandemic in individuals aged 19–64

| Variables | | Influenza vaccinated (before pandemic) | | Influenza vaccinated (during pandemic) | | Ratio of ORs (95% CI), Influenza vaccinated (during pandemic) compared to Influenza vaccinated (before pandemic) | |
| --- | --- | --- | --- | --- | --- | --- | --- |
|  |  | Weighted OR (95% CI) | P–value | Weighted OR (95% CI) | P–value | Ratio of ORs (95% CI) | P–value |
| 19–64 years old | | | | | | | |
| Age | 19–29 | 1.00 (ref) |  | 1.00 (ref) |  |  |  |
|  | 30–49 | **1.34 (1.28 to 1.41)** | **<.001** | **1.34 (1.29 to 1.39)** | **<.001** | 1.00 (0.94 to 1.06) | 0.883 |
|  | 50–64 | **1.66 (1.58 to 1.75)** | **<.001** | **1.49 (1.43 to 1.55)** | **<.001** | **0.90 (0.84 to 0.96)** | **<.001** |
| Sex | Male | 1.00 (ref) |  | 1.00 (ref) |  |  |  |
|  | Female | **1.38 (1.34 to 1.43)** | **<.001** | **1.35 (1.31 to 1.39)** | **<.001** | 0.97 (0.93 to 1.02) | 0.234 |
| Region of residence | Urban | 1.00 (ref) |  | 1.00 (ref) |  |  |  |
|  | Rural | **1.17 (1.14 to 1.21)** | **<.001** | 1.03 (1.00 to 1.06) | 0.090 | **0.88 (0.84 to 0.92)** | **<.001** |
| Education | High school or lower education | 1.00 (ref) |  | 1.00 (ref) |  |  |  |
|  | College or higher education | 1.01 (0.98 to 1.04) | 0.717 | **0.97 (0.95 to 1.00)** | **0.030** | 0.97 (0.93 to 1.01) | 0.090 |
| Household income | Lowest and second quartile income | 1.00 (ref) |  | 1.00 (ref) |  |  |  |
|  | Third and highest quartile income | **0.85 (0.82 to 0.87)** | **<.001** | 1.00 (0.97 to 1.04) | 0.813 | **1.19 (1.14 to 1.24)** | **<.001** |
| BMI | Underweight or normal | 1.00 (ref) |  | 1.00 (ref) |  |  |  |
|  | Overweight or obese | **1.08 (1.06 to 1.11)** | **<.001** | **1.03 (1.01 to 1.06)** | **0.009** | **0.95 (0.92 to 0.99)** | **0.007** |
| Economic activity status | Yes | 1.00 (ref) |  | 1.00 (ref) |  |  |  |
|  | No | 0.97 (0.67 to 1.41) | 0.878 | **1.29 (1.24 to 1.34)** | **<.001** | 1.33 (0.91 to 1.93) | 0.136 |
| Occupational categories | Employer or self–employed | 1.00 (ref) |  | 1.00 (ref) |  |  |  |
|  | Salaried | **1.28 (1.24 to 1.33)** | **<.001** | **1.35 (1.31 to 1.40)** | **<.001** | **1.05 (1.01 to 1.11)** | **0.029** |
|  | Unemployed | 1.34 (0.92 to 1.94) | 0.127 | **1.35 (1.31 to 1.40)** | **<.001** | 1.01 (0.70 to 1.47) | 0.950 |
| Marital status | Yes | 1.00 (ref) |  | 1.00 (ref) |  |  |  |
|  | No | **0.64 (0.62 to 0.66)** | **<.001** | **0.64 (0.62 to 0.66)** | **<.001** | 1.00 (0.96 to 1.05) | 0.944 |
| Smoking status | Non–smoker | 1.00 (ref) |  | 1.00 (ref) |  |  |  |
|  | Current and ex–smoker | **0.82 (0.79 to 0.85)** | **<.001** | **0.81 (0.79 to 0.83)** | **<.001** | 0.99 (0.94 to 1.04) | 0.642 |
| Alcohol intake | Non–drinker | 1.00 (ref) |  | 1.00 (ref) |  |  |  |
|  | Drinker | **0.89 (0.87 to 0.91)** | **<.001** | **0.94 (0.91 to 0.96)** | **<.001** | 1.05 (1.01 to 1.09) | 0.006 |
| Hypertension | Yes | 1.00 (ref) |  | 1.00 (ref) |  |  |  |
|  | No | **0.74 (0.72 to 0.77)** | **<.001** | **0.74 (0.72 to 0.77)** | **<.001** | 1.00 (0.95 to 1.05) | 1.000 |
| Diabetes | Yes | 1.00 (ref) |  | 1.00 (ref) |  |  |  |
|  | No | **0.76 (0.72 to 0.79)** | **<.001** | **0.76 (0.73 to 0.80)** | **<.001** | 1.01 (0.95 to 1.08) | 0.757 |
| Sufficient physical activity | Yes | 1.00 (ref) |  | 1.00 (ref) |  |  |  |
|  | No | **0.90 (0.88 to 0.92)** | **<.001** | **0.91 (0.89 to 0.93)** | **<.001** | 1.01 (0.97 to 1.05) | 0.626 |
| General health status | Third and highest quartile | 1.00 (ref) |  | 1.00 (ref) |  |  |  |
|  | Second quartile | **1.06 (1.02 to 1.10)** | **0.003** | 1.02 (0.98 to 1.05) | 0.359 | 0.96 (0.91 to 1.01) | 0.127 |
|  | Lowest quartile | **1.09 (1.05 to 1.13)** | **<.001** | **1.04 (1.00 to 1.07)** | **0.046** | 0.95 (0.90 to 1.00) | 0.047 |
| Depression | Yes | 1.00 (ref) |  | 1.00 (ref) |  |  |  |
|  | No | 0.99 (0.92 to 1.07) | 0.846 | 1.04 (0.98 to 1.11) | 0.223 | 1.05 (0.95 to 1.16) | 0.353 |
| Unmet needs for healthcare services | Insufficient | 1.00 (ref) |  | 1.00 (ref) |  |  |  |
|  | Sufficient | **1.21 (1.15 to 1.27)** | **<.001** | **1.20 (1.14 to 1.27)** | **<.001** | 1.00 (0.92 to 1.07) | 0.914 |

Abbreviations: BMI, body mass index; CI, confidence interval; OR, odds ratio.

The multivariable logistic regression was adjusted by age (19–29, 30–49, 50–64, 65–74, and ≥75 years), sex, region of residence (urban and rural), education (elementary school or lower education, middle school, high school, and college or higher education), household income (lowest, second, third, and highest quartile), BMI (underweight, normal weight, overweight, and obese), economic activity status, occupational categories (employer or self–employed, salaried, and unemployed), marital status, smoking status (non–smoker, ex–smoker, and smoker), alcohol intake (non–drinker, drinker), hypertension, diabetes, sufficient physical activity, general health status (third and highest, second, and lowest quartile), depression, and unmet needs for healthcare services (insufficient and sufficient).

The numbers in bold indicate significant differences (p<0.05).

**Table S4.** Adjusted and weighted odds ratios for influenza before pandemic and SARS–CoV–2 vaccination in individuals aged ≥65

| Variables | | Influenza vaccinated (before pandemic) | | SARS–CoV–2 vaccinated | | Ratio of ORs (95% CI), SARS–CoV–2 vaccinated compared to influenza vaccinated (before pandemic) | |
| --- | --- | --- | --- | --- | --- | --- | --- |
|  |  | Weighted OR (95% CI) | P–value | Weighted OR (95% CI) | P–value | Ratio of ORs (95% CI) | P–value |
| Over 65 years old | | | | | | | |
| Age | 65–74 | 1.00 (ref) |  | 1.00 (ref) |  |  |  |
|  | ≥75 | **1.93 (1.82 to 2.06)** | **<.001** | **1.14 (1.04 to 1.24)** | **0.004** | **0.59 (0.53 to 0.65)** | **<.001** |
| Sex | Male | 1.00 (ref) |  | 1.00 (ref) |  |  |  |
|  | Female | **1.17 (1.06 to 1.29)** | **0.002** | **1.45 (1.27 to 1.67)** | **<.001** | **1.24 (1.05 to 1.47)** | **0.011** |
| Region of residence | Urban | 1.00 (ref) |  | 1.00 (ref) |  |  |  |
|  | Rural | **1.08 (1.02 to 1.15)** | **0.012** | 1.09 (1.00 to 1.19) | 0.052 | 1.01 (0.90 to 1.12) | 0.907 |
| Education | High school or lower education | 1.00 (ref) |  | 1.00 (ref) |  |  |  |
|  | College or higher education | **0.70 (0.63 to 0.77)** | **<.001** | **0.83 (0.72 to 0.95)** | **0.007** | **1.19 (1.00 to 1.41)** | **0.045** |
| Household income | Lowest and second quartile income | 1.00 (ref) |  | 1.00 (ref) |  |  |  |
|  | Third and highest quartile income | **0.89 (0.83 to 0.96)** | **0.002** | 1.08 (0.96 to 1.20) | 0.203 | **1.21 (1.06 to 1.38)** | **0.006** |
| BMI | Underweight or normal | 1.00 (ref) |  | 1.00 (ref) |  |  |  |
|  | Overweight or obese | **1.13 (1.07 to 1.19)** | **<.001** | **1.29 (1.19 to 1.40)** | **<.001** | **1.15 (1.04 to 1.27)** | **0.006** |
| Economic activity status | Yes | 1.00 (ref) |  | 1.00 (ref) |  |  |  |
|  | No | 0.79 (0.31 to 2.02) | 0.616 | **0.81 (0.69 to 0.95)** | **0.011** | 1.03 (0.40 to 2.69) | 0.949 |
| Occupational categories | Employer or self–employed | 1.00 (ref) |  | 1.00 (ref) |  |  |  |
|  | Salaried | 1.07 (0.98 to 1.17) | 0.139 | **1.92 (1.53 to 2.42)** | **<.001** | **1.80 (1.40 to 2.30)** | **<.001** |
|  | Unemployed | 1.39 (0.54 to 3.57) | 0.493 | **1.92 (1.53 to 2.42)** | **<.001** | 1.38 (0.52 to 3.65) | 0.516 |
| Marital status | Yes | 1.00 (ref) |  | 1.00 (ref) |  |  |  |
|  | No | **0.70 (0.66 to 0.75)** | **<.001** | **0.67 (0.61 to 0.74)** | **<.001** | 0.96 (0.86 to 1.08) | 0.503 |
| Smoking status | Non–smoker | 1.00 (ref) |  | 1.00 (ref) |  |  |  |
|  | Current and ex–smoker | **0.83 (0.76 to 0.91)** | **<.001** | 0.99 (0.87 to 1.14) | 0.911 | **1.20 (1.01 to 1.41)** | **0.034** |
| Alcohol intake | Non–drinker | 1.00 (ref) |  | 1.00 (ref) |  |  |  |
|  | Drinker | **0.92 (0.87 to 0.99)** | **0.015** | **1.92 (1.69 to 2.17)** | **<.001** | **2.07 (1.80 to 2.39)** | **<.001** |
| Hypertension | Yes | 1.00 (ref) |  | 1.00 (ref) |  |  |  |
|  | No | **0.61 (0.57 to 0.64)** | **<.001** | **0.84 (0.78 to 0.92)** | **<.001** | **1.39 (1.26 to 1.54)** | **<.001** |
| Diabetes | Yes | 1.00 (ref) |  | 1.00 (ref) |  |  |  |
|  | No | **0.77 (0.72 to 0.83)** | **<.001** | 0.92 (0.83 to 1.01) | 0.067 | **1.18 (1.05 to 1.33)** | **0.006** |
| Sufficient physical activity | Yes | 1.00 (ref) |  | 1.00 (ref) |  |  |  |
|  | No | **0.86 (0.80 to 0.91)** | **<.001** | **0.58 (0.51 to 0.67)** | **<.001** | **0.68 (0.59 to 0.79)** | **<.001** |
| General health status | Third and highest quartile | 1.00 (ref) |  | 1.00 (ref) |  |  |  |
|  | Second quartile | **1.19 (1.09 to 1.30)** | **<.001** | 0.92 (0.79 to 1.08) | 0.291 | **0.77 (0.65 to 0.92)** | **0.005** |
|  | Lowest quartile | **1.11 (1.04 to 1.18)** | **0.002** | **0.55 (0.50 to 0.61)** | **<.001** | **0.50 (0.44 to 0.56)** | **<.001** |
| Depression | Yes | 1.00 (ref) |  | 1.00 (ref) |  |  |  |
|  | No | 1.10 (0.96 to 1.26) | 0.170 | **1.40 (1.23 to 1.60)** | **<.001** | **1.28 (1.06 to 1.54)** | **0.011** |
| Unmet needs for healthcare services | Insufficient | 1.00 (ref) |  | 1.00 (ref) |  |  |  |
|  | Sufficient | **1.79 (1.59 to 2.00)** | **<.001** | **1.96 (1.70 to 2.27)** | **<.001** | 1.10 (0.91 to 1.32) | 0.323 |

Abbreviations: BMI, body mass index; CI, confidence interval; OR, odds ratio.

The multivariable logistic regression was adjusted by age (19–29, 30–49, 50–64, 65–74, and ≥75 years), sex, region of residence (urban and rural), education (elementary school or lower education, middle school, high school, and college or higher education), household income (lowest, second, third, and highest quartile), BMI (underweight, normal weight, overweight, and obese), economic activity status, occupational categories (employer or self–employed, salaried, and unemployed), marital status, smoking status (non–smoker, ex–smoker, and smoker), alcohol intake (non–drinker, drinker), hypertension, diabetes, sufficient physical activity, general health status (third and highest, second, and lowest quartile), depression, and unmet needs for healthcare services (insufficient and sufficient).

The numbers in bold indicate significant differences (p<0.05).

**Table S5.** Adjusted and weighted odds ratios for influenza vaccination before and during the pandemic in individuals aged ≥65

| Variables | | Influenza vaccinated (before pandemic) | | Influenza vaccinated (during pandemic) | | Ratio of ORs (95% CI), Influenza vaccinated (during pandemic) compared to Influenza vaccinated (before pandemic) | |
| --- | --- | --- | --- | --- | --- | --- | --- |
|  |  | Weighted OR (95% CI) | P–value | Weighted OR (95% CI) | P–value | Ratio of ORs (95% CI) | P–value |
| Over 65 years old | | | | | | | |
| Age | 65–74 | 1.00 (ref) |  | 1.00 (ref) |  |  |  |
|  | ≥75 | **1.93 (1.82 to 2.06)** | **<.001** | **1.76 (1.67 to 1.86)** | **<.001** | **0.91 (0.84 to 0.99)** | **0.030** |
| Sex | Male | 1.00 (ref) |  | 1.00 (ref) |  |  |  |
|  | Female | **1.17 (1.06 to 1.29)** | **0.002** | **1.19 (1.10 to 1.29)** | **<.001** | 1.02 (0.90 to 1.15) | 0.802 |
| Region of residence | Urban | 1.00 (ref) |  | 1.00 (ref) |  |  |  |
|  | Rural | **1.08 (1.02 to 1.15)** | **0.012** | **1.15 (1.09 to 1.22)** | **<.001** | 1.06 (0.98 to 1.15) | 0.149 |
| Education | High school or lower education | 1.00 (ref) |  | 1.00 (ref) |  |  |  |
|  | College or higher education | **0.70 (0.63 to 0.77)** | **<.001** | **0.71 (0.66 to 0.76)** | **<.001** | 1.02 (0.90 to 1.15) | 0.763 |
| Household income | Lowest and second quartile income | 1.00 (ref) |  | 1.00 (ref) |  |  |  |
|  | Third and highest quartile income | **0.89 (0.83 to 0.96)** | **0.002** | **0.92 (0.87 to 0.98)** | **0.009** | 1.04 (0.94 to 1.14) | 0.458 |
| BMI | Underweight or normal | 1.00 (ref) |  | 1.00 (ref) |  |  |  |
|  | Overweight or obese | **1.13 (1.07 to 1.19)** | **<.001** | **1.14 (1.08 to 1.20)** | **<.001** | 1.01 (0.94 to 1.09) | 0.835 |
| Economic activity status | Yes | 1.00 (ref) |  | 1.00 (ref) |  |  |  |
|  | No | 0.79 (0.31 to 2.02) | 0.616 | **1.15 (1.07 to 1.25)** | **<.001** | 1.47 (0.57 to 3.78) | 0.426 |
| Occupational categories | Employer or self–employed | 1.00 (ref) |  | 1.00 (ref) |  |  |  |
|  | Salaried | 1.07 (0.98 to 1.17) | 0.139 | **1.26 (1.15 to 1.38)** | **<.001** | **1.18 (1.04 to 1.34)** | **0.012** |
|  | Unemployed | 1.39 (0.54 to 3.57) | 0.493 | **1.26 (1.15 to 1.38)** | **<.001** | 0.90 (0.35 to 2.33) | 0.834 |
| Marital status | Yes | 1.00 (ref) |  | 1.00 (ref) |  |  |  |
|  | No | **0.70 (0.66 to 0.75)** | **<.001** | **0.70 (0.66 to 0.74)** | **<.001** | 1.00 (0.92 to 1.09) | 0.922 |
| Smoking status | Non–smoker | 1.00 (ref) |  | 1.00 (ref) |  |  |  |
|  | Current and ex–smoker | **0.83 (0.76 to 0.91)** | **<.001** | **0.92 (0.85 to 0.99)** | **0.028** | 1.10 (0.98 to 1.25) | 0.104 |
| Alcohol intake | Non–drinker | 1.00 (ref) |  | 1.00 (ref) |  |  |  |
|  | Drinker | **0.92 (0.87 to 0.99)** | **0.015** | **0.89 (0.84 to 0.94)** | **<.001** | 0.96 (0.88 to 1.05) | 0.372 |
| Hypertension | Yes | 1.00 (ref) |  | 1.00 (ref) |  |  |  |
|  | No | **0.61 (0.57 to 0.64)** | **<.001** | **0.69 (0.66 to 0.73)** | **<.001** | **1.14 (1.06 to 1.22)** | **<.001** |
| Diabetes | Yes | 1.00 (ref) |  | 1.00 (ref) |  |  |  |
|  | No | **0.77 (0.72 to 0.83)** | **<.001** | **0.85 (0.80 to 0.90)** | **<.001** | 1.09 (1.00 to 1.20) | 0.058 |
| Sufficient physical activity | Yes | 1.00 (ref) |  | 1.00 (ref) |  |  |  |
|  | No | **0.86 (0.80 to 0.91)** | **<.001** | **0.85 (0.80 to 0.91)** | **<.001** | 0.99 (0.91 to 1.09) | 0.881 |
| General health status | Third and highest quartile | 1.00 (ref) |  | 1.00 (ref) |  |  |  |
|  | Second quartile | **1.19 (1.09 to 1.30)** | **<.001** | **1.14 (1.05 to 1.22)** | **0.001** | 0.95 (0.85 to 1.07) | 0.424 |
|  | Lowest quartile | **1.11 (1.04 to 1.18)** | **0.002** | 0.98 (0.93 to 1.04) | 0.540 | **0.89 (0.81 to 0.97)** | **0.007** |
| Depression | Yes | 1.00 (ref) |  | 1.00 (ref) |  |  |  |
|  | No | 1.10 (0.96 to 1.26) | 0.170 | **1.32 (1.19 to 1.46)** | **<.001** | **1.20 (1.01 to 1.42)** | **0.037** |
| Unmet needs for healthcare services | Insufficient | 1.00 (ref) |  | 1.00 (ref) |  |  |  |
|  | Sufficient | **1.79 (1.59 to 2.00)** | **<.001** | **1.79 (1.62 to 1.99)** | **<.001** | 1.00 (0.86 to 1.17) | 0.955 |

Abbreviations: BMI, body mass index; CI, confidence interval; OR, odds ratio.

The multivariable logistic regression was adjusted by age (19–29, 30–49, 50–64, 65–74, and ≥75 years), sex, region of residence (urban and rural), education (elementary school or lower education, middle school, high school, and college or higher education), household income (lowest, second, third, and highest quartile), BMI (underweight, normal weight, overweight, and obese), economic activity status, occupational categories (employer or self–employed, salaried, and unemployed), marital status, smoking status (non–smoker, ex–smoker, and smoker), alcohol intake (non–drinker, drinker), hypertension, diabetes, sufficient physical activity, general health status (third and highest, second, and lowest quartile), depression, and unmet needs for healthcare services (insufficient and sufficient).

The numbers in bold indicate significant differences (p<0.05).

**Table S6.** Adjusted and weighted odds ratios for influenza during pandemic and SARS–CoV–2 vaccination in individuals aged 19–64

| Variables | | Influenza vaccinated (during pandemic) | | SARS–CoV–2 vaccinated | | Ratio of ORs (95% CI), SARS–CoV–2 vaccinated compared to Influenza vaccinated (during pandemic) | |
| --- | --- | --- | --- | --- | --- | --- | --- |
|  |  | Weighted OR (95% CI) | P–value | Weighted OR (95% CI) | P–value | Ratio of ORs (95% CI) | P–value |
| 19–64 years old | | | | | | | |
| Age | 19–29 | 1.00 (ref) |  | 1.00 (ref) |  |  |  |
|  | 30–49 | **1.34 (1.29 to 1.39)** | **<.001** | **1.06 (1.01 to 1.11)** | **0.015** | **0.79 (0.75 to 0.84)** | **<.001** |
|  | 50–64 | **1.49 (1.43 to 1.55)** | **<.001** | **6.37 (5.97 to 6.80)** | **<.001** | **4.28 (3.97 to 4.62)** | **<.001** |
| Sex | Male | 1.00 (ref) |  | 1.00 (ref) |  |  |  |
|  | Female | **1.35 (1.31 to 1.39)** | **<.001** | **1.30 (1.25 to 1.35)** | **<.001** | 0.96 (0.92 to 1.01) | 0.129 |
| Region of residence | Urban | 1.00 (ref) |  | 1.00 (ref) |  |  |  |
|  | Rural | 1.03 (1.00 to 1.06) | 0.090 | 1.03 (0.98 to 1.08) | 0.198 | 1.00 (0.95 to 1.06) | 0.866 |
| Education | High school or lower education | 1.00 (ref) |  | 1.00 (ref) |  |  |  |
|  | College or higher education | **0.97 (0.95 to 1.00)** | **0.030** | **1.34 (1.29 to 1.39)** | **<.001** | **1.38 (1.32 to 1.45)** | **<.001** |
| Household income | Lowest and second quartile income | 1.00 (ref) |  | 1.00 (ref) |  |  |  |
|  | Third and highest quartile income | 1.00 (0.97 to 1.04) | 0.813 | **1.41 (1.35 to 1.47)** | **<.001** | **1.41 (1.33 to 1.48)** | **<.001** |
| BMI | Underweight or normal | 1.00 (ref) |  | 1.00 (ref) |  |  |  |
|  | Overweight or obese | **1.03 (1.01 to 1.06)** | **0.009** | **1.10 (1.06 to 1.14)** | **<.001** | **1.06 (1.02 to 1.11)** | **0.003** |
| Economic activity status | Yes | 1.00 (ref) |  | 1.00 (ref) |  |  |  |
|  | No | **1.29 (1.24 to 1.34)** | **<.001** | **0.68 (0.66 to 0.71)** | **<.001** | **0.53 (0.50 to 0.55)** | **<.001** |
| Occupational categories | Employer or self–employed | 1.00 (ref) |  | 1.00 (ref) |  |  |  |
|  | Salaried | **1.35 (1.31 to 1.40)** | **<.001** | **1.21 (1.15 to 1.27)** | **<.001** | **0.89 (0.84 to 0.95)** | **<.001** |
|  | Unemployed | **1.35 (1.31 to 1.40)** | **<.001** | **1.21 (1.15 to 1.27)** | **<.001** | **0.89 (0.84 to 0.95)** | **<.001** |
| Marital status | Yes | 1.00 (ref) |  | 1.00 (ref) |  |  |  |
|  | No | **0.64 (0.62 to 0.66)** | **<.001** | 0.99 (0.94 to 1.03) | 0.519 | **1.54 (1.46 to 1.62)** | **<.001** |
| Smoking status | Non–smoker | 1.00 (ref) |  | 1.00 (ref) |  |  |  |
|  | Current and ex–smoker | **0.81 (0.79 to 0.83)** | **<.001** | **0.88 (0.84 to 0.91)** | **<.001** | **1.09 (1.03 to 1.14)** | **0.001** |
| Alcohol intake | Non–drinker | 1.00 (ref) |  | 1.00 (ref) |  |  |  |
|  | Drinker | **0.94 (0.91 to 0.96)** | **<.001** | **1.35 (1.31 to 1.40)** | **<.001** | **1.45 (1.39 to 1.51)** | **<.001** |
| Hypertension | Yes | 1.00 (ref) |  | 1.00 (ref) |  |  |  |
|  | No | **0.74 (0.72 to 0.77)** | **<.001** | **0.80 (0.76 to 0.85)** | **<.001** | **1.08 (1.01 to 1.15)** | **0.028** |
| Diabetes | Yes | 1.00 (ref) |  | 1.00 (ref) |  |  |  |
|  | No | **0.76 (0.73 to 0.80)** | **<.001** | 0.97 (0.90 to 1.05) | 0.505 | **1.27 (1.16 to 1.40)** | **<.001** |
| Sufficient physical activity | Yes | 1.00 (ref) |  | 1.00 (ref) |  |  |  |
|  | No | **0.91 (0.89 to 0.93)** | **<.001** | **0.78 (0.76 to 0.81)** | **<.001** | **0.86 (0.83 to 0.90)** | **<.001** |
| General health status | Third and highest quartile | 1.00 (ref) |  | 1.00 (ref) |  |  |  |
|  | Second quartile | 1.02 (0.98 to 1.05) | 0.359 | 0.98 (0.93 to 1.03) | 0.348 | 0.96 (0.90 to 1.02) | 0.202 |
|  | Lowest quartile | **1.04 (1.00 to 1.07)** | **0.046** | **0.81 (0.77 to 0.85)** | **<.001** | **0.78 (0.74 to 0.83)** | **<.001** |
| Depression | Yes | 1.00 (ref) |  | 1.00 (ref) |  |  |  |
|  | No | 1.04 (0.98 to 1.11) | 0.223 | 1.00 (0.92 to 1.09) | 0.986 | 0.96 (0.86 to 1.07) | 0.469 |
| Unmet needs for healthcare services | Insufficient | 1.00 (ref) |  | 1.00 (ref) |  |  |  |
|  | Sufficient | **1.20 (1.14 to 1.27)** | **<.001** | **1.14 (1.06 to 1.22)** | **<.001** | 0.95 (0.87 to 1.04) | 0.233 |

Abbreviations: BMI, body mass index; CI, confidence interval; OR, odds ratio.

The multivariable logistic regression was adjusted by age (19–29, 30–49, 50–64, 65–74, and ≥75 years), sex, region of residence (urban and rural), education (elementary school or lower education, middle school, high school, and college or higher education), household income (lowest, second, third, and highest quartile), BMI (underweight, normal weight, overweight, and obese), economic activity status, occupational categories (employer or self–employed, salaried, and unemployed), marital status, smoking status (non–smoker, ex–smoker, and smoker), alcohol intake (non–drinker, drinker), hypertension, diabetes, sufficient physical activity, general health status (third and highest, second, and lowest quartile), depression, and unmet needs for healthcare services (insufficient and sufficient).

The numbers in bold indicate significant differences (p<0.05).

**Table S7.** Adjusted and weighted odds ratios for influenza during pandemic and SARS–CoV–2 vaccination in individuals aged ≥65

| Variables | | Influenza vaccinated (during pandemic) | | SARS–CoV–2 vaccinated | | Ratio of ORs (95% CI), SARS–CoV–2 vaccinated compared to Influenza vaccinated (during pandemic) | |
| --- | --- | --- | --- | --- | --- | --- | --- |
|  |  | Weighted OR (95% CI) | P–value | Weighted OR (95% CI) | P–value | Ratio of ORs (95% CI) | P–value |
| Over 65 years old | | | | | | | |
| Age | 65–74 | 1.00 (ref) |  | 1.00 (ref) |  |  |  |
|  | ≥75 | **1.76 (1.67 to 1.86)** | **<.001** | **1.14 (1.04 to 1.24)** | **0.004** | **0.64 (0.58 to 0.71)** | **<.001** |
| Sex | Male | 1.00 (ref) |  | 1.00 (ref) |  |  |  |
|  | Female | **1.19 (1.10 to 1.29)** | **<.001** | **1.45 (1.27 to 1.67)** | **<.001** | **1.22 (1.04 to 1.43)** | **0.013** |
| Region of residence | Urban | 1.00 (ref) |  | 1.00 (ref) |  |  |  |
|  | Rural | **1.15 (1.09 to 1.22)** | **<.001** | 1.09 (1.00 to 1.19) | 0.052 | 0.95 (0.85 to 1.05) | 0.304 |
| Education | High school or lower education | 1.00 (ref) |  | 1.00 (ref) |  |  |  |
|  | College or higher education | **0.71 (0.66 to 0.76)** | **<.001** | **0.83 (0.72 to 0.95)** | **0.007** | 1.17 (1.00 to 1.36) | 0.054 |
| Household income | Lowest and second quartile income | 1.00 (ref) |  | 1.00 (ref) |  |  |  |
|  | Third and highest quartile income | **0.92 (0.87 to 0.98)** | **0.009** | 1.08 (0.96 to 1.20) | 0.203 | **1.16 (1.03 to 1.32)** | **0.018** |
| BMI | Underweight or normal | 1.00 (ref) |  | 1.00 (ref) |  |  |  |
|  | Overweight or obese | **1.14 (1.08 to 1.20)** | **<.001** | **1.29 (1.19 to 1.40)** | **<.001** | **1.14 (1.03 to 1.25)** | **0.008** |
| Economic activity status | Yes | 1.00 (ref) |  | 1.00 (ref) |  |  |  |
|  | No | **1.15 (1.07 to 1.25)** | **<.001** | **0.81 (0.69 to 0.95)** | **0.011** | **0.70 (0.59 to 0.84)** | **<.001** |
| Occupational categories | Employer or self–employed | 1.00 (ref) |  | 1.00 (ref) |  |  |  |
|  | Salaried | **1.26 (1.15 to 1.38)** | **<.001** | **1.92 (1.53 to 2.42)** | **<.001** | **1.53 (1.19 to 1.96)** | **<.001** |
|  | Unemployed | **1.26 (1.15 to 1.38)** | **<.001** | **1.92 (1.53 to 2.42)** | **<.001** | **1.53 (1.19 to 1.96)** | **<.001** |
| Marital status | Yes | 1.00 (ref) |  | 1.00 (ref) |  |  |  |
|  | No | **0.70 (0.66 to 0.74)** | **<.001** | **0.67 (0.61 to 0.74)** | **<.001** | 0.96 (0.86 to 1.07) | 0.438 |
| Smoking status | Non–smoker | 1.00 (ref) |  | 1.00 (ref) |  |  |  |
|  | Current and ex–smoker | **0.92 (0.85 to 0.99)** | **0.028** | 0.99 (0.87 to 1.14) | 0.911 | 1.08 (0.93 to 1.26) | 0.324 |
| Alcohol intake | Non–drinker | 1.00 (ref) |  | 1.00 (ref) |  |  |  |
|  | Drinker | **0.89 (0.84 to 0.94)** | **<.001** | **1.92 (1.69 to 2.17)** | **<.001** | **2.16 (1.88 to 2.48)** | **<.001** |
| Hypertension | Yes | 1.00 (ref) |  | 1.00 (ref) |  |  |  |
|  | No | **0.69 (0.66 to 0.73)** | **<.001** | **0.84 (0.78 to 0.92)** | **<.001** | **1.22 (1.11 to 1.35)** | **<.001** |
| Diabetes | Yes | 1.00 (ref) |  | 1.00 (ref) |  |  |  |
|  | No | **0.85 (0.80 to 0.90)** | **<.001** | 0.92 (0.83 to 1.01) | 0.067 | 1.08 (0.97 to 1.21) | 0.176 |
| Sufficient physical activity | Yes | 1.00 (ref) |  | 1.00 (ref) |  |  |  |
|  | No | **0.85 (0.80 to 0.91)** | **<.001** | **0.58 (0.51 to 0.67)** | **<.001** | **0.69 (0.59 to 0.80)** | **<.001** |
| General health status | Third and highest quartile | 1.00 (ref) |  | 1.00 (ref) |  |  |  |
|  | Second quartile | **1.14 (1.05 to 1.22)** | **0.001** | 0.92 (0.79 to 1.08) | 0.291 | **0.81 (0.68 to 0.96)** | **0.017** |
|  | Lowest quartile | 0.98 (0.93 to 1.04) | 0.540 | **0.55 (0.50 to 0.61)** | **<.001** | **0.56 (0.50 to 0.63)** | **<.001** |
| Depression | Yes | 1.00 (ref) |  | 1.00 (ref) |  |  |  |
|  | No | **1.32 (1.19 to 1.46)** | **<.001** | **1.40 (1.23 to 1.60)** | **<.001** | 1.06 (0.90 to 1.26) | 0.466 |
| Unmet needs for healthcare services | Insufficient | 1.00 (ref) |  | 1.00 (ref) |  |  |  |
|  | Sufficient | **1.79 (1.62 to 1.99)** | **<.001** | **1.96 (1.70 to 2.27)** | **<.001** | 1.09 (0.91 to 1.31) | 0.332 |

Abbreviations: BMI, body mass index; CI, confidence interval; OR, odds ratio.

The multivariable logistic regression was adjusted by age (19–29, 30–49, 50–64, 65–74, and ≥75 years), sex, region of residence (urban and rural), education (elementary school or lower education, middle school, high school, and college or higher education), household income (lowest, second, third, and highest quartile), BMI (underweight, normal weight, overweight, and obese), economic activity status, occupational categories (employer or self–employed, salaried, and unemployed), marital status, smoking status (non–smoker, ex–smoker, and smoker), alcohol intake (non–drinker, drinker), hypertension, diabetes, sufficient physical activity, general health status (third and highest, second, and lowest quartile), depression, and unmet needs for healthcare services (insufficient and sufficient).

The numbers in bold indicate significant differences (p<0.05).

**Table S8.** Association between the various risk factors and vaccine uptake for influenza before and during the pandemic, and SARS–CoV–2 based on data obtained from the KCHS

| Variables | | Weighted OR (95% CI) | P–value | Weighted OR (95% CI) | P–value | Weighted OR (95% CI) | P–value |
| --- | --- | --- | --- | --- | --- | --- | --- |
|  |  | Influenza vaccinated (before pandemic) | | Influenza vaccinated (during pandemic) | | SARS–CoV–2 vaccinated | |
| Age | 19–64 | 1.00 (ref) |  | 1.00 (ref) |  | 1.00 (ref) |  |
|  | ≥65 | **10.06 (9.69 to 10.44)** | **<.001** | **6.75 (6.53 to 6.98)** | **<.001** | **4.34 (4.12 to 4.57)** | **<.001** |
| Sex | Male | 1.00 (ref) |  | 1.00 (ref) |  | 1.00 (ref) |  |
|  | Female | **1.39 (1.34 to 1.43)** | **<.001** | **1.35 (1.31 to 1.39)** | **<.001** | **1.31 (1.27 to 1.36)** | **<.001** |
| Region of residence | Urban | 1.00 (ref) |  | 1.00 (ref) |  | 1.00 (ref) |  |
|  | Rural | **1.18 (1.14 to 1.21)** | **<.001** | **1.05 (1.03 to 1.08)** | **<.001** | 1.04 (1.00 to 1.09) | 0.051 |
| Education | High school or lower education | 1.00 (ref) |  | 1.00 (ref) |  | 1.00 (ref) |  |
|  | College or higher education | **0.90 (0.88 to 0.93)** | **<.001** | **0.90 (0.88 to 0.92)** | **<.001** | **0.88 (0.85 to 0.91)** | **<.001** |
| Household income | Lowest and second quartile income | 1.00 (ref) |  | 1.00 (ref) |  | 1.00 (ref) |  |
|  | Third and highest quartile income | **0.82 (0.80 to 0.85)** | **<.001** | **0.96 (0.93 to 0.99)** | **0.003** | **1.16 (1.12 to 1.21)** | **<.001** |
| BMI | Underweight or normal | 1.00 (ref) |  | 1.00 (ref) |  | 1.00 (ref) |  |
|  | Overweight or obese | **1.08 (1.06 to 1.11)** | **<.001** | **1.04 (1.02 to 1.06)** | **<.001** | **1.09 (1.06 to 1.12)** | **<.001** |
| Economic activity status | Yes | 1.00 (ref) |  | 1.00 (ref) |  | 1.00 (ref) |  |
|  | No | 0.97 (0.70 to 1.35) | 0.840 | **1.22 (1.18 to 1.26)** | **<.001** | **0.73 (0.69 to 0.76)** | **<.001** |
| Occupational categories | Employer or self–employed | 1.00 (ref) |  | 1.00 (ref) |  | 1.00 (ref) |  |
|  | Salaried | **1.22 (1.18 to 1.26)** | **<.001** | **1.31 (1.27 to 1.35)** | **<.001** | 1.01 (0.96 to 1.06) | 0.749 |
|  | Unemployed | 1.27 (0.91 to 1.77) | 0.157 | **1.31 (1.27 to 1.35)** | **<.001** | 1.01 (0.96 to 1.06) | 0.749 |
| Marital status | Yes | 1.00 (ref) |  | 1.00 (ref) |  | 1.00 (ref) |  |
|  | No | **0.58 (0.57 to 0.60)** | **<.001** | **0.57 (0.56 to 0.59)** | **<.001** | **0.69 (0.67 to 0.71)** | **<.001** |
| Smoking status | Non–smoker | 1.00 (ref) |  | 1.00 (ref) |  | 1.00 (ref) |  |
|  | Current and ex–smoker | **0.84 (0.81 to 0.87)** | **<.001** | **0.84 (0.81 to 0.86)** | **<.001** | **0.93 (0.89 to 0.96)** | **<.001** |
| Alcohol intake | Non–drinker | 1.00 (ref) |  | 1.00 (ref) |  | 1.00 (ref) |  |
|  | Drinker | **0.87 (0.85 to 0.89)** | **<.001** | **0.91 (0.89 to 0.93)** | **<.001** | **1.22 (1.18 to 1.26)** | **<.001** |
| Hypertension | Yes | 1.00 (ref) |  | 1.00 (ref) |  | 1.00 (ref) |  |
|  | No | **0.66 (0.64 to 0.68)** | **<.001** | **0.69 (0.67 to 0.71)** | **<.001** | **0.57 (0.55 to 0.60)** | **<.001** |
| Diabetes | Yes | 1.00 (ref) |  | 1.00 (ref) |  | 1.00 (ref) |  |
|  | No | **0.75 (0.72 to 0.78)** | **<.001** | **0.77 (0.74 to 0.80)** | **<.001** | **0.75 (0.71 to 0.80)** | **<.001** |
| Sufficient physical activity | Yes | 1.00 (ref) |  | 1.00 (ref) |  | 1.00 (ref) |  |
|  | No | **0.90 (0.88 to 0.93)** | **<.001** | **0.92 (0.90 to 0.94)** | **<.001** | **0.79 (0.76 to 0.82)** | **<.001** |
| General health status | Third and highest quartile | 1.00 (ref) |  | 1.00 (ref) |  | 1.00 (ref) |  |
|  | Second quartile | **1.10 (1.06 to 1.14)** | **<.001** | **1.06 (1.02 to 1.09)** | **<.001** | **1.11 (1.06 to 1.16)** | **<.001** |
|  | Lowest quartile | **1.15 (1.12 to 1.19)** | **<.001** | **1.07 (1.04 to 1.11)** | **<.001** | **0.84 (0.81 to 0.88)** | **<.001** |
| Depression | Yes | 1.00 (ref) |  | 1.00 (ref) |  | 1.00 (ref) |  |
|  | No | 1.05 (0.98 to 1.13) | 0.143 | **1.12 (1.06 to 1.19)** | **<.001** | **1.20 (1.12 to 1.29)** | **<.001** |
| Unmet needs for healthcare services | Insufficient | 1.00 (ref) |  | 1.00 (ref) |  | 1.00 (ref) |  |
|  | Sufficient | **1.29 (1.23 to 1.36)** | **<.001** | **1.28 (1.22 to 1.35)** | **<.001** | **1.24 (1.16 to 1.32)** | **<.001** |

Abbreviations: BMI, body mass index; CI, confidence interval; KCHS, Korea Community Health Service; OR, odds ratio.

The numbers in bold indicate significant differences (p<0.05).
